# Assessing the level of digital maturity of enterprises in the Central and Eastern European countries using the MCDM and Shannon’s entropy methods

**DOI:** 10.1371/journal.pone.0253965

**Published:** 2021-07-06

**Authors:** Jarosław Brodny, Magdalena Tutak

**Affiliations:** Silesian University of Technology, Gliwice, Poland; Gonbad Kavous University, ISLAMIC REPUBLIC OF IRAN

## Abstract

The process of global economic digitalization is a natural stage of evolutionary changes resulting from a dynamic development of information and communication technologies. Having appreciated the importance and advantages of digital transformation, individual countries began to strive to introduce it as soon as possible. In this context, it is important to study the level of digital maturity in Central and Eastern Europe, where the level of digitization is relatively low. This article assesses the level of digital readiness of enterprises in these countries based on 14 determinants characterizing the most important areas of the digitalization process. The research was carried out for 11 countries from the region, both for all and manufacturing enterprises. Multi-criteria analysis aimed at assessing the digital maturity of countries were performed using the Multi-Criteria Decision-Making methods (the TOPSIS, MOORA, VIKOR), and entropy methods for delineating the weights of the determinants. In order to obtain an unambiguous assessment of the determined digital maturity, the mean-rank method was applied. The method of multidimensional scaling allowed for the analysis of similarities between the countries in question. The results showed that the level of digital maturity in the Central and Eastern Europe countries varies greatly and is lower than in other European Union countries.

## 1. Introduction

The digital transformation of the global economy is a process closely related to the idea of Industry 4.0 [1]. Activities connected with economic digitization significantly affect the activities of individual companies. In order to meet the growing competition and technological changes, these companies have to significantly change their strategies, including, in many cases, the way they operate and their business profile [2]. It is also significantly affected by a very dynamically changing economic and social environment, including both customer preferences and expectations [3].

All these factors result in a growing transformation in the digitization of manufacturing, service and commercial enterprises observed for several years around the world. These changes are the consequence of technological progress, which forces a new approach to production itself and the organization and management of enterprises (new business models), as well as to social (corporate social responsibility) and environmental (sustainable closed-loop economy) problems [4–6].

In addition to innovative technical solutions and the new organization of production processes, digitalization causes major changes in the labor market and social awareness. From the economic point of view, the transformation of the labor market related to Industry 4.0 is of particular importance. Employees’ competences in the area of ubiquitous digitalization and the resulting economic changes are now becoming most sought-after and desired. Significant changes are also taking place in the area of consumer expectations, which concern the subject, location and object, but also the entire consumption process [7]. Therefore, we are witnessing the emergence of a new digital society in the sphere of production, services, trade, and consumption.

The digital transformation process is now a key tool to improve the efficiency and competitiveness of enterprises, which are constantly looking for new solutions to optimize their production. Technologies associated with digital transformation, which are widely used in enterprises, include mainly technologies related to the integration of different systems and the creation of digitally controlled networks of autonomous machines and sensors, the Internet of things, as well as a number of other solutions (e.g., 3D printing). In general, these processes are based on changes in information systems, and thanks to solutions such as cloud computing, big data analytics or Internet of things, it is possible to access any information at any time and from any place in the world. As a result, it is possible, economically viable and flexible to manufacture custom-made or small series products, developed according to the strict needs of the customer [8–10].

Most of the studies and analyses clearly indicate that investments in the digital economy are fully justified and profitable. At the same time, the experience of recent years shows that the basis for the development of the global economy in the coming years will be its digitization.

The importance of the issue of digitization of enterprises in the EU countries is evidenced by its inclusion in the political strategy for 2019–2024. This strategy is based on several key priorities. One of them is to bring Europe up to the digital age. It also aims, through digital transformation, to raise living standards in Europe and achieve its climate neutrality by 2050 [11]. In 2021, the EU also launched the Digital Europe Program [12] with a budget of €8.1 billion. This program is to financially support the digital transformation of European societies and economies.

The need for this program is visible in many EU countries, especially those located in Central and Eastern Europe (CEE). In the case of these countries (Poland, the Czech Republic, Slovakia, Slovenia, Romania, Hungary, Croatia, Latvia, Lithuania, Estonia, and Bulgaria), the actual innovative activities are still limited, and expenditure on research and development (R&D) in relation to GDP is two times lower than the EU average [13].

These statistics clearly show that the digitization of the CEE countries requires both huge expenditure and significant economic and social changes. According to available research [14], only 13% of entities operating in these countries declare that they have a digital transformation strategy for their economy or are at an advanced stage of its implementation. These results are really low and confirm that digitization processes in this part of Europe are far behind. Many factors that influence this state can be distinguished. One of the main reasons is that the implementation time of the free market economy in these countries took too short, which resulted in the lack of management experience and adequate financial resources. Also, the relatively low investment in research means that innovation in these countries, despite significant growth in recent years, is still low versus the "old" EU economies. However, the growing understanding of the need to introduce changes related to digital transformation, increasing economic activity and European solidarity create a great opportunity for the region to catch up with these delays. Moreover, the growing social awareness, including mainly the young generation, and the desire to build a modern and profitable knowledge-based economy are also great opportunities for success in this area.

In this context, it is fully justified to conduct research aimed at assessing the digital maturity of enterprises in the CEE countries. The diagnosis of the current state of the economies of these countries will allow for their assessment and pointing out similarities and differences between them. The general measure of digital maturity in this case will be the number of processes, including production processes, implemented using modern technologies [15]. The concept of digital maturity is defined in various ways, e.g., the Cambridge Dictionary [16] states that maturity is "a very advanced or developed form or state”, while the Oxford Dictionary [17] defines maturity as "the state, fact, or period of being mature".

Based on different approaches to defining this state, it can be concluded that digital maturity is a certain state of social and economic awareness that enables enterprises to effectively implement digital technologies to achieve their goals. According to such an understanding of digital maturity, this paper presents the results of the analysis, on the basis of which its level was assessed in the enterprises of the CEE countries.

In order to obtain reliable and objective results, a number of methods and criteria were adopted. The analysis involved the set of 14 indicators, representing eight areas related to the digitization of enterprises and the area related to the digital skills of staff. The adoption of such a large number of indicators, characterizing different areas of economic and social activities, provides an opportunity for a comprehensive assessment of the state of enterprises in terms of digital maturity. In addition to adopting indicators that characterize the most important areas related to digitalization, it is very important to adopt appropriate methods for their analysis. In the presented study, the Multi-Criteria Decision-Making (MCDM) methods were applied, which allow for both ranking and evaluation to be made in a holistic manner. These methods present a multidisciplinary approach and make it possible, through the use of numerical techniques, to solve a multi-criteria decision-making problem.

Three multi-criteria decision-making methods such as Technique for Order of Preference by Similarity to Ideal Solution (TOPSIS), Multi-Objective Optimization Method by Ratio Analysis (MOORA) and Vise Kriterijumska Optimizacija I Kompromisno Resenje (VIKOR) were used to rank and evaluate the level of digital maturity of enterprises in the CEE, being the part of the EU. These methods were used because they have the same input data and are all based on the same normalization procedure. In addition, in these methods the solution procedure does not change regardless of the number of decision criteria and alternatives.

It was assumed that the adoption of three different methods, which show some differences in the approach to multi-criteria analysis, would enable a broader and deeper analysis of the digital maturity of these countries. The use of these methods was also intended to show whether the choice of research method affected the results and, if so, to what extent. In order to obtain an unambiguous assessment of the digital maturity of each country, the mean-rank method was additionally applied, averaging the results obtained.

The main objective of the research was to assess the level of digital maturity of all and manufacturing enterprises in the CEE countries. The utilitarian goal of the research was to develop guidelines for future actions that can be taken to increase the level of digitization of enterprises in the countries in question.

The subject and wide scope of the research as well as the applied analytical tools make the work a new and original approach to assessing the digital maturity of the CEE countries. This type of research has not been carried out so far. In addition, several factors can also be identified that prove the originality of this work in relation to existing studies.

The first concerns the selection of as many as 14 determinants of digital maturity of enterprises in the areas most relevant for this process, namely: big data, cloud computing, 3D printing, robotization, integration of internal processes, integration with customers/suppliers, supply chain management, Internet of things, artificial intelligence, and digital skills. Such a broad and comprehensive approach to the study of this issue has not been undertaken before. The second factor is the choice of countries for the study. The countries of Central and Eastern Europe are a very important part of Europe due to many factors, including their demographic potential and geo-political location. This is of great significance for the future of the EU. Therefore, the development of this part of Europe must be accelerated so that the whole EU can achieve its stated and very ambitious economic and climate goals. The third factor concerns the use of modern, and so far unused in such a system, analytical methods to make the ranking and assessment of the level of digital maturity of enterprises in a given group of countries. This involves the application of the Multi-Criteria Decision-Making (MCDM) methodologies, including the TOPSIS, MOORA and VIKOR methods, as well as the entropy method for delineating the weights of the determinants. Moreover, the use of the mean-rank method as well as the method of multidimensional scaling and STRESS function makes the presented analysis a completely new analytical approach, which increases its scientific value and gives credibility to the obtained results. Another factor proving the originality of the work is the analysis of differences between the CEE countries in terms of digital maturity of enterprises. For this purpose, the multidimensional scaling method was used to determine the differences between these countries and compare them with other EU countries.

## 2. Brief literature review

### 2.1. Digital transformation and Industry 4.0

In the field of digital transformation related to Industry 4.0, many scientific studies and examples of practical applications can be found. The presented review refers only to the most relevant works. It also includes publications related to the assessment of digital maturity of enterprises.

The concept of Industry 4.0 was launched in Germany in 2011 [18], and the term refers to the dynamically occurring changes due to the practical application of modern technologies in social and economic life.

In the United States, on the other hand, the concept of Industry 4.0 is commonly known as the Internet Industry of Things, advanced manufacturing or smart manufacturing. Although the term "smart industry" was originally used only in the United States, it is now used worldwide both in industry and scientific units [19].

According to Dilberoglu et al. [20] and Mosterman i Zander [21], Industry 4.0 is an integrated set of intelligent manufacturing systems and advanced information technologies based on integrated sets of software systems. However, according to Guoping et al. [22], Industry 4.0 is a set of technologies based on the digitization and interconnection of all production units present in an economic system. Therefore, it can be assumed that Industry 4.0 is the integration of various systems through the use of digital resources, including intelligent ones, which by communicating with one another can support and make certain decisions as well as perform various operations with much less human involvement than before [23]. The basic technologies of Industry 4.0 along with literature references are presented in Table 1.

**Table 1:** Industry 4.0 technologies.

Industry 4.0 technologies	Sources
Big data; Autonomous robot; Internet of Things; Additive mfg.; Artificial intelligence	[24]
Big data; Autonomous robot; Horizontal and Vertical System Integration; Internet of Things; Cybersecurity; Cloud computing; Additive mfg.; Augmented reality	[22]
Internet of Things; Additive mfg.; Augmented reality	[25]
Big data; Horizontal and Vertical System Integration; Internet of Things; Cloud computing; Augmented reality; Cyber-physical system	[26]
Big data; Autonomous robot; Simulation; Horizontal and Vertical System Integration; Internet of Things; Cybersecurity; Cloud computing; Additive mfg.; Augmented reality; Cyber-physical system; Artificial intelligence	[27]
Horizontal and Vertical System Integration; Internet of Things; Cloud computing; Cyber-physical system	[28]
Horizontal and Vertical System Integration Industrial Internet of Things; Cybersecurity; Cyber Physical Systems; Horizontal and Vertical System Integration; Augmented reality	[29]
Big data; Internet of Things; Cloud computing	[30]
Big data; Autonomous robot; Internet of Things; Cloud computing; (H) Additive mfg.; Cyber-physical system; Artificial intelligence	[31]
Additive manufacturing; Virtual reality/augmented reality; Robotics; Internet of Things; Cybersecurity (Blockchain); Big data; Artificial intelligence	[32]

In terms of publications on assessing the level of maturity and/or readiness for the Industry 4.0 technologies, it should be noted that their number has been growing recently.

One study [33] proposes a novel and innovative model to assess the degree of readiness of a manufacturing company to implement the Industry 4.0 technologies. Based on this model, a tool was developed to identify actions needed to increase the degree of readiness of companies to implement the Industry 4.0 principles and practices. The proposed approach was inspired by the framework developed by SAE to identify and measure best practices for implementing lean manufacturing in companies [34].

Leineweber et al. [35] presented a method for assessing the digital maturity of enterprises using more than 40 evaluation criteria from the areas of technology, human resources and organization. Sarvari et al. [36] showed a technology development roadmap for the Industry 4.0 transformation in an enterprise to facilitate the planning and implementation process of this concept. The map developed helps to understand each move and what decisions need to be made, as well as who should make them and when during the digital transformation in an enterprise. Schumacher et al. [15] proposed a model to assess the maturity of Industry 4.0 among industrial enterprises in the domain of discrete manufacturing. This model includes 9 dimensions to which 62 items were assigned to evaluate the maturity of Industry 4.0. The dimensions "Products", "Customers", "Operations" and "Technology" were created to assess the basic factors. In addition, the dimensions "Strategy", "Leadership", "Management", "Culture" and "People" allowed for the inclusion of organizational aspects in this assessment.

Branco et al. [37] examined factors characterizing readiness for Industry 4.0. in the manufacturing companies of the EU countries. The analysis proved that the existence of digital infrastructure combined with analytical capabilities to handle large data sets makes it possible to achieve a high level of readiness of manufacturing companies for Industry 4.0. Basl and Doucek [38] developed a metamodel to assess an organization’s digital readiness for Industry 4.0. It ranks selected maturity models and readiness indicators against each other, while identifying areas with potential for further research. Bibby and Dehe [39] developed an assessment framework for maturity for Industry 4.0. in a central company to later compare it with 12 organizations in its supply network. Enterprise assessment focuses on three dimensions: smart factory, people and culture, and strategy. Akdil et al. [40] proposed an Industry 4.0 maturity assessment model and a questionnaire to assess this level. The model covers different application areas of Industry 4.0 such as smart finance, smart marketing and human resources. In order to determine a company’s maturity level for Industry 4.0, four stages were included: "None", "Existence", "Survival" and "Maturity". The questions in each criterion had a 4-stage scale to assess the level of digital maturity–from 0:"None" to 3:"Maturity". Kuruczleki et al. [41] determined the readiness of the EU-28 countries for the fourth industrial revolution by creating a readiness index of Industry 4.0 consisting of eight indicators: total intramural R&D expenditure, gross domestic expenditure on R&D, community trade mark applications, community design applications, total R&D personnel and researchers, tertiary educational attainment, ICT specialists, digital single market–promoting e-commerce for businesses, enterprises selling online. Lizarralde et al. [42] developed a maturity model for machine tool companies. This maturity model is evaluation and identification tool for the areas of the organization where specific development is required. Liebrecht et al. [43] developed a methodology to support decision making of industrial companies for the Industry 4.0 method application in a production environment. Tortora et al. [44] conducted a survey on the level of readiness of Italian small and medium-sized enterprises to introduce the idea of Industry 4.0. The results showed that these companies have limited and insufficient knowledge of Industry 4.0 technologies. More than 50% of the respondents demonstrated a basic or low level of knowledge of the technologies in question. Saad et al. [45] adapted and used the Smart SME Technology Readiness Assessment (SSTRA) methodology to investigate the level of technology readiness of these companies to implement Industry 4.0 based on smart production planning and control. De Carolis et al. [46] proposed a model to study the digital maturity of a company. The assessment was carried out in 5 areas: design and engineering as well as production, quality, maintenance and logistics management. This approach considers all relevant aspects related to the idea of Industry 4.0. Lin et al. [47] used a model based on the Singapore Smart Industry Readiness Index by the Economic Development Board to assess digital maturity. This model enables companies to conduct self-assessment in order to systematically and comprehensively adapt to Industry 4.0. The assessment is conducted in three Building Blocks: Process, Technology and Organization, and 16 dimensions in total. Jung et al. [48] introduced the Smart Manufacturing System Readiness Level (SMSRL) to measure a company’s digital readiness in four dimensions: organizational maturity, IT maturity, performance measurement, and information connectivity. The model enables small and medium-sized manufacturing companies to assess their readiness to implement the necessary technologies that can help them in their digital transformation.

A summary of selected models for assessing digital maturity and readiness, along with their brief description, is presented in Table 2.

**Table 2:** Maturity and readiness models.

Maturity or Readiness Models	Description	Source
A maturity model for Industry 4.0 Readiness	This model allows for the assessment of digital maturity in 6 dimensions (Strategy, Leadership, Customer, Products, Operations, Culture, People, Governance, and Technology). The level is rated on a Likert scale (from 1 = "not important"; to 4 = "very important").	[15]
The Degree of readiness for the implementation of Industry 4.0	This model assesses the level of digital maturity across 8 dimensions (Internet of Things, Big Data, Cloud Computing, Cyber Physical Systems, Collaborative Robots, Additive Manufacturing, Augmented Reality, Artificial Intelligence), and on a 6-point scale from 1 (Embryonic) to 6 (Ready).	[33]
An Overview of a Smart Manufacturing System Readiness Assessment	To assess the level of digital maturity, this model uses 4 dimensions (Organizational maturity, IT maturity, Performance Management maturity, Information Connectivity maturity) and a 6-point scale from 1 (Not performed) to 6 (Optimizing).	[48]
The Connected Enterprise Maturity Model	The model is based on a 5-stage approach for implementing Industry 4.0 in an organization in terms of IT readiness (Assessment; Secure and upgraded network controls; Defined and organized working data capital (WDC); Analytics; Collaboration). The model introduces 4 dimensions of assessment in which the focus is mainly on technology.	[49]
IMPULS–Industry 4.0 readiness	The model allows for the assessment of digital maturity. There are 6 levels of this maturity: Outsiders; Beginners; Intermediate; Experienced; Expert; Top performers) and the assessment is conducted in 6 dimensions: (Strategy & Organization, Smart Factory, Smart Operations, Smart Products, Data-driven Services, and Employees).	[50]
Digital readiness for Industry 4.0	This model allows for the assessment of the level of digital maturity in 6 dimensions: (Business Models; Product & Service; Portfolio Market & Customer Access; Value Chains & Processes; IT Architecture; Compliance, Legal, Risk, Security & Tax; Organization & Culture).	[51]
SIMMI 4.0	This model allows for the assessment of the level of digital maturity in 3 dimensions (Vertical Integration, Horizontal Integration, Cross-sectional Technology Criteria). It also distinguishes 5 stages of maturity (basic digitization; cross-sectional digitization; horizontal and vertical digitization; full digitization; and optimized full digitization).	[52]
Towards a Smart Manufacturing Maturity Model for SMEs	This model assesses the level of digital maturity in 5 dimensions. (Finance, People, Strategy, Process, and Product), on a 5-point scale from 1 (Novice) to 5 (Expert).	[53]
The Logistics 4.0 Maturity Model	To make assessment, this model uses 3 dimensions and a 5-point scale from 1 (Ignoring) to 5 (Integrated).	[54]
A Smartness Assessment Framework for Smart Factories Using Analytic Network Process	This model allows for the assessment of the level of digital maturity in 4 dimensions (criteria) (Leadership, Process, System & Automation, Performance) and in 10 subcriteria. A 5-point scale is used for the assessment from 1 (Checking) to 5 (Autonomy).	[55]

### 2.2. Application of MCDM methods

MCDM methods are used to solve complex decision-making issues in various areas. They are also adopted in issues related to Industry 4.0 and the digitization of economies and societies [56–62] (Table 3). However, so far these methods have not been used to assess the digital maturity of enterprises.

**Table 3:** Summary of applications of MCDM methods to solve different problems.

Problem	Methods	Source
Evaluation of Digital Marketing Technologies	Analytic Hierarchy Process (AHP) Complex Proportional Assessment (COPRAS)	[56]
Multistage performance modelling in digital marketing management	TOPSIS	[57]
Streamlining of digital marketing management activities	Fuzzy ANP	[58]
Decision-making model for identifying appropriate technologies for effective digital transformation in Automotive Supplier Industry	TOPSIS	[59]
Optimization of the problem of supplier selection and order allocation in the era of Industry 4.0	Step method (STEM)	[60]
Prioritization of public services for digitalization	Fuzzy Z-AHP Z-WASPAS	[61]

### 2.3. Research gaps

The literature search shows that the topic of digitalization is current and refers to various aspects of this process. Research on the effects of its implementation seems to be fully justified both for the current assessment and for the prospects of development.

Its results also indicate that in the vast majority of the presented studies, on the assessment of digital readiness and/or maturity, no importance weighting was taken into account for the adopted criteria or dimensions (factors) [33,39,63–65].

Only a small number of these works include weights for the adopted factors for assessment [15,50,66].

The variety of factors taken into account in these studies, and their different impact on the processes studied, shows the relevance of including weights in multi-criteria analyses regarding digitization.

The search also shows that the multiple criteria-decision making methods have not been used so far to assess digital readiness and/or maturity of enterprises. Also, the presented assessments failed to include developing countries, which are undoubtedly the countries of Central and Eastern Europe.

Therefore, there is a research gap in terms of studying digital maturity in the CEE companies, which is due to the lack of this type of studies. That is why, the conducted literature search fully rationalizes examining such a research subject. Also, the application of computational methods from the area of multi-criteria decision-making methodologies such as TOPSIS, MOORA and VIKOR methods, makes this study a new approach to this type of analysis. It allows for a broader look at the research and increases its both reliability and credibility. In turn, the obtained knowledge creates wide possibilities of interpretation, inference and formulation of recommendations.

## 3. Materials and methods

**Fig 1:**
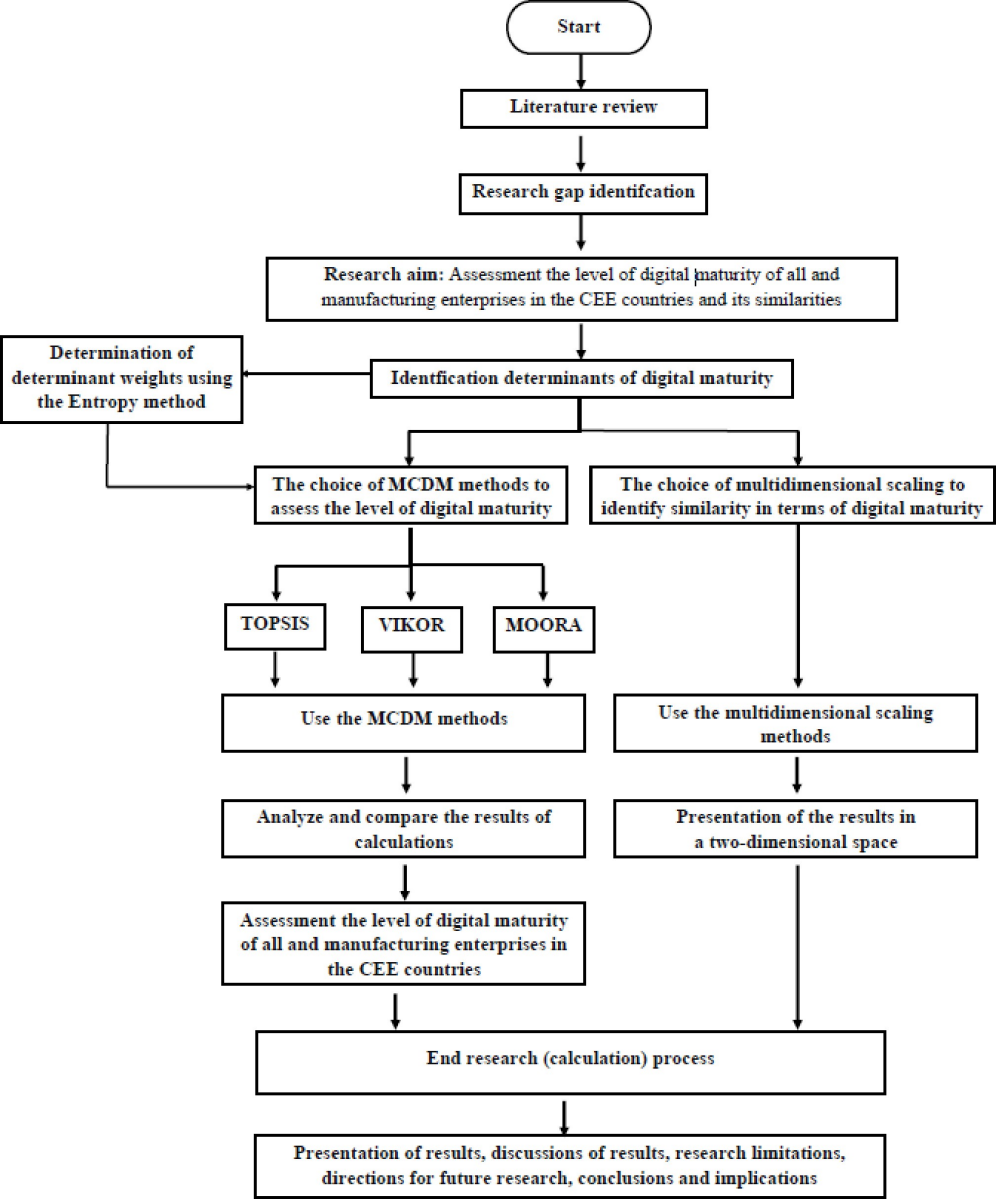
Research scheme.

In order to assess the level of digital maturity of companies in general and manufacturing companies in CEE countries, as well as to analyze their similarity, the research was conducted based on a set of 14 determinants of the digitization process. MCDM and multidimensional scaling methods were used for analysis. The scheme of the research procedure is shown in Fig 1. Subsection 3.2 discusses the adopted determinants of digital maturity used for the research, and subsection 3.3 characterizes the applied research methods.

### 3.1. Area of research

**Fig 2:**
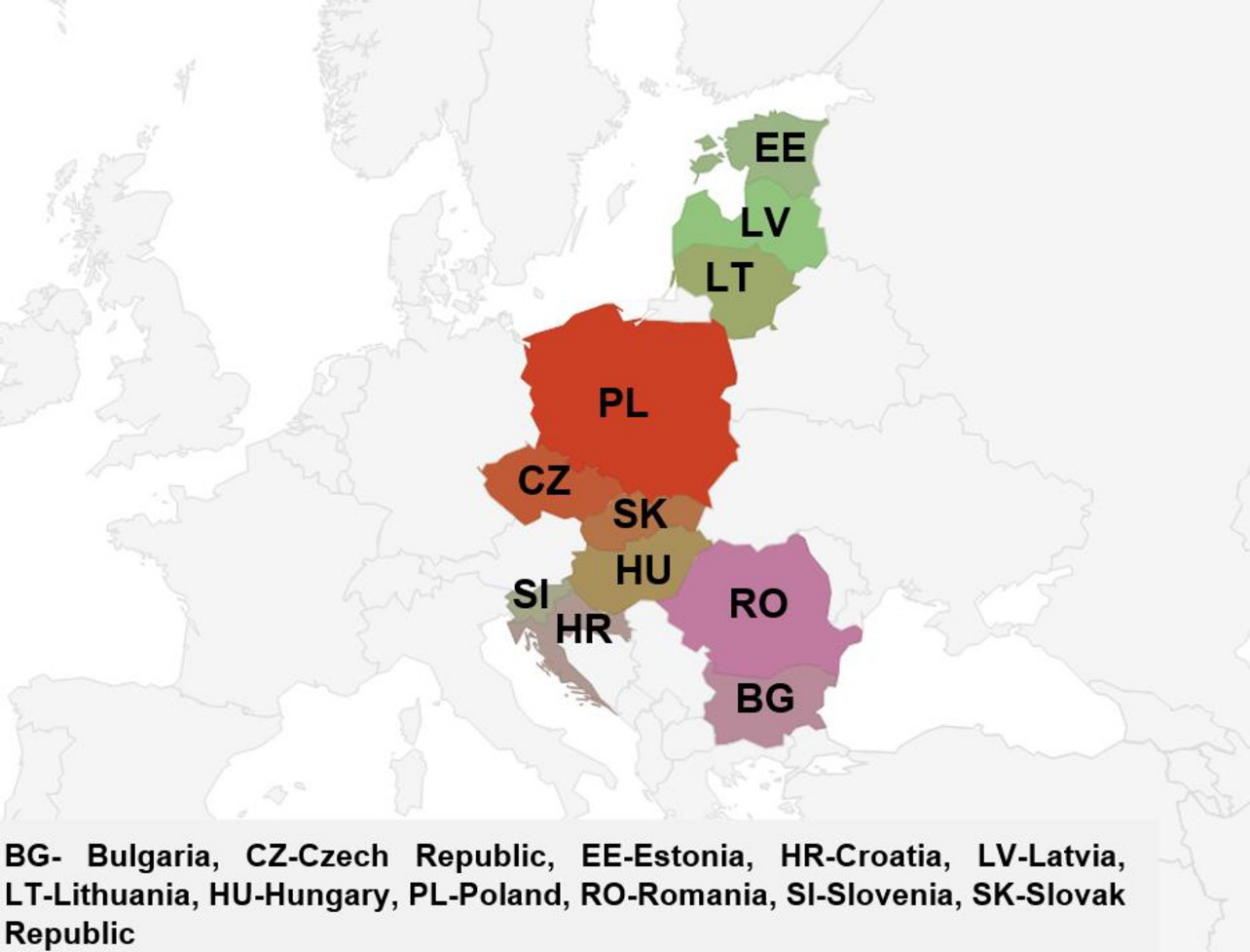
Countries of Central and Eastern Europe belonging to the European Union.

The European Union is a community of 27 member states, 11 of which belong to the countries of Central and Eastern Europe (CEE) (Fig 2). These countries include Poland, the Czech Republic, Slovakia, Slovenia, Bulgaria, Romania, Croatia, Lithuania, Latvia, Estonia, and Hungary (Fig 2, Table 4). All of them have common cultural and historical roots. They also share a common past as socialist buffer states under the USSR or as its constituent parts, namely the Soviet republics. The term Central and Eastern European countries is a composite of two terms for this part of Europe–geographical (Central) and political (Eastern). Basic information about these countries is presented in Table 4.

**Table 4:** Basic information about the CEE countries belonging to the EU (own elaboration based on [67]).

Countries	Group of countries	Year of joining the EU	GDP value (2020) million euro	GDP per capita (2020) euro per capita	Population	Area, km^2^
Poland	Visegrad group	2004	521 514.5	13 600	37 958 138	312,685
Czech Republic		2004	213 589.1	19 960	10 693 939	78,866
Slovakia		2004	91 104.8	16 680	5 457 873	49,036
Hungary		2004	135 529.4	13 900	9 769 526	93,030
Lithuania	Baltic countries	2004	48 794.2	17 460	2 794 090	65,300
Latvia		2004	29 334.0	15 430	1 907 675	64,589
Estonia		2004	27 166.9	20 440	1 328 976	45,339
Slovenia	Former Yugoslavia	2004	46 297.2	22 010	2 095 861	20,273
Croatia		2013	49 104.1	12 130	4 058 165	56,594
Bulgaria	Balkan countries	2007	60 642.7	8 750	6 951 482	110,994
Romania		2007	217 820.6	11 270	19 328 838	238,397

### 3.2. Materials

In order to assess the level of digital maturity of enterprises, including manufacturing enterprises in the CEE countries, data from the Eurostat database were used [67]. They concern the use of information and communication technologies (ICT) in enterprises and digital skills. On the basis of this data, a set of determinants was determined to rank and assess the level of digital maturity of enterprises in the ECC countries and to analyze their similarities.

In order to assess the level of digital readiness of all enterprises and manufacturing enterprises in the CEE countries, it was necessary to select appropriate determinants. Based on the literature review, 9 areas that have a decisive influence on the level of digital readiness of manufacturing companies were selected. Eight areas are related to the Industry 4.0 technologies and one to digital skills of employees.

For these areas, 14 determinants of digital maturity were adopted, the description of which is presented in Table 5. The selected determinants of digital maturity meet the postulate of relevance to the topic related to Industry 4.0 (they refer to the main pillars of Industry 4.0). They are characterized by simplicity in construction, ease of interpretation of results, availability and high quality of data, which are updated on an ongoing basis, as well as they are comparable at an international level.

**Table 5:** The adopted determinants characterizing the digitalization processes of enterprises in the CEE countries.

Area of Industry 4.0	Determinant	Explanation
**Big data analytics**	Analysis of big data from smart devices or sensors	Big data analytics technologies currently play a huge role in the introduction of all kinds of innovative solutions. The development of digital technologies is based on the analysis of large data sets. The digitalization of enterprises cannot be effectively carried out without the development of this area of research.
	Analysis of big data from the geolocation of portable devices	
**Artificial intelligence**	Analysis of big data internally using machine learning	Artificial intelligence makes it possible to make the most of the data processed by components connected through the IoT. Based on telemetry data, an AI-equipped device can control the entire system of interconnected, synchronized machines working together through IoT, control individual devices, and even make autonomous decisions.
**Cloud computing**	Purchase of cloud computing services used over the Internet	The use of cloud computing brings many benefits to enterprises. First of all, it lowers operating costs (reduces the cost of maintaining IT infrastructure) and increases data security. Cloud technologies enable, among others, the storage of data, applications, programs, as well as their operation from any place in the world (only Internet access is required). Access to the latest technologies and the ease of use are serious arguments for their use.
	Purchase of high CC services	
**3D printing**	Use of 3D printing	The 3D printing technology is currently one of the most rapidly growing fields related to the digitalization of the global economy. It is used by many companies in the processes of prototyping, as well as toolmaking, small batch production, and other activities. The 3D printing technology allows companies to complete the full cycle of product manufacturing in a short time, which, in many cases, is their competitive advantage.
**Robotics**	Use of industrial or service robots	The robotization of production processes involving the replacement of human activities with machines is the quintessential process of the digitization of the economy. The introduction of robotization is associated with quite high costs, but the advantages of this process are enormous. It is worth mentioning only the efficiency and quality of production which can be in this case at a level virtually unattainable by employees.
**Integration of internal processes**	Enterprises which have ERP software package to share information between different functional areas	ERP applications (systems) in companies are designed to facilitate the flow of information and the possibility of horizontal and vertical integration. The comprehensiveness of these systems and their functionality, usually based on large data sets, gives great opportunities to optimize production processes in an enterprise. This is mainly due to the integration of processes related to business planning, the purchase of goods and services, marketing processes, sales, enterprise-consumer relations, company finances, and human resources.
	Enterprises using software solutions like Customer Relationship Management	
**Integration with customers/suppliers, supply chain management**	Enterprises sending eInvoices, suitable for automated processing	Supply chain management includes all activities related to the exchange of information between an enterprise and its suppliers and/or customers. The digitalization of this area of the companies’ activities is crucial for the optimization of their operations. It can be assumed that the digitalization of business processes in an enterprise is a prerequisite for its development in the digital economy.
**Internet of Things**	Use of interconnected devices or systems that can be monitored or remotely controlled via the Internet (Internet of Things)	The Internet of things plays one of the key roles in today’s businesses. IoT combines information technology with operational technology, which refers to the networking of processes and industrial control systems (ICS) (human-machine interfaces, production supervision software or programmable logic controllers). The interaction of Internet of Things technology with operational technology ensures, among other things, system consistency in terms of automation and optimization, better data availability, etc. IoT systems are also used to locate autonomous vehicles or workers and to control environmental factors. This technology requires the use of data. Devices that make up the IoT can provide information to artificial intelligence algorithms that analyze data in real time.
	Use of smart meters, smart lamps, smart thermostats to optimize energy consumption in the enterprise’s premises	
	Use of sensors or RFID tags to monitor or automate production processes, to manage logistics, to track the movement of products	
**Digital skills (ICT training)**	Enterprises that provided training to develop/upgrade ICT skills of their personnel	The introduction of digital technologies is associated with the need to build a digital society. Currently, digital skills are considered to be a basic condition that determines the possibility of developing a digital economy. They require continuous upgrading of skills by employees so that, with the development and implementation of new technologies, there is no phenomenon of digital exclusion.

### 3.3. Methods

A set of multi-criteria decision-making methods was used to assess the level of digital maturity of enterprises in the CEE countries. These methods include: TOPSIS method, VIKOR method and MOORA method. Brief characteristics of these methods are presented in this section.

#### 3.3.1 The VIKOR method

The VIKOR method belongs to the methods of multi-criteria decision optimization and is based on the concept of measuring the distance of the studied variant from the ideal scenario [68]. This method introduces the so-called ranking index, based on the measurement of distance from the ideal solution. This index is an extension of the aggregate function theory in the compromise programming method [69,70]. Individual variants belonging to set *A (a*_*1*_, *a*_*2*_,… *a*_*m*_*)* and evaluated by *n* criteria are described by the factor *fij*, which is the weight of the variant *a*_*j*_ with respect to the criterion *n*_*i*_.

The output parameter for the analysis conducted by the VIKOR method is the *Lp*-metric distance, determined from Eq (1):

$$L_{pj}={\left\{\sum_{i=1}^{n}\left[w_{i}\frac{\left(x_{i}^{*}-x_{ij}\right)}{{\left(x_{i}^{*}-x_{i}^{-}\right)}^{p}}\right]\right\}}^{\frac{1}{p}};1\leq p\leq \infty ,j=1,2,\ldots ,m$$ (1)

where: $x_{i}^{*}$ and $x_{i}^{-}$ are the best and worst values of all criterion functions for all alternatives from set *A (a*_*1*_, *a*_*2*_,… *a*_*m*_*)*; *n* is the number of criteria.

The algorithm of the VIKOR method to determine the compromise ranking is as follows:

- to construct the decision matrix, according to Eq (2):

$$X={\left[x_{ij}\right]}_{m\times n}=\left[\begin{array}{ccc} x_{11} & \cdots & x_{1n}\\ \vdots & \ddots & \vdots \\ x_{m1} & \cdots & x_{mn} \end{array}\right]$$ (2)

where: *x*_*ij*_∈ℝ

- to construct the normalized decision matrix, according to Eq (3):

$$x_{ij}=\frac{x_{ij}}{\sqrt{\sum_{i=1}^{m}x_{ij}^{2}}};\;\forall i,j$$ (3)

- to determine the best $x_{i}^{*}$ and worst $x_{i}^{-}$ values for all criterion functions i = 1, 2,…n. If the i-th criterion represents profit (the higher the better), then $x_{i}^{*}={max}_{j}\;x_{ij}$ and $x_{i}^{-}={min}_{j}\;x_{ij}$; if the i-th criterion represents cost (the lower the better), then $x_{i}^{*}={min}_{j}\;x_{ij}$ and $x_{i}^{-}={max}_{j}\;x_{ij}$;
- to calculate S_j_ and R_j_ values forming the ranking measure, from the following relationships:

$$L_{1j}=S_{j}=\sum_{i=1}^{n}w_{i}\frac{\left(x_{i}^{*}-x_{j}\right)}{\left(x_{i}^{*}-x_{i}^{-}\right)}$$ (4)

$$L_{\infty j}=R_{j}=max\left[w_{i}\frac{\left(x_{i}^{*}-x_{ij}\right)}{\left(x_{i}^{*}-x_{i}^{-}\right)}\right]$$ (5)

where: *w*_*i*_ weight of the *i*-th criterion; *S*_*i*_ and *R*_*i*_ represent the utylity measure and the regret measure.

The solution obtained by *min*_*j*_*S*_*j*_ is with the maximum group utility (’majority’ rule), and the solution obtained by *min*_*j*_*R*_j_ is with the minimum individual regret of the ’opponent’

- To calculate the VIKOR–Q_j_ index value (6):

$$Q_{j}=\frac{v(S_{j}-S^{*})}{\left(S^{-}-S^{*}\right)}+(1-v)\frac{\left(R_{j}-R_{*}\right)}{\left(R^{-}R^{*}\right)}$$ (6)

where:

$$S^{*}={min}_{j}S_{j};\;S^{-}={max}_{j}S_{j};\;R^{*}={min}_{j}{SR}_{j};\;R^{-}={max}_{j}R_{j}$$ (7)

where: *v* is the measure of strategy weight (takes value from 0 to 1); *S*_*j*_ and *R*_*j*_ are calculated in Step 3 and are introduced as the weight of strategy of ’the majority of criteria’ (or ’the maximum group utility’). In the present study, *v* = 0.5.

- to create the ranking of alternatives according to the value of *Q*_*j*_.

The alternative with the smallest VIKOR value is referred to as the best (*Q*_*minimum*_).

#### 3.3.2 The TOPSIS method

The TOPSIS method is one of the most popular methods used for solving multicriteria discrete tasks [71,72]. The considered decision variants are compared with abstract weighted reference solutions: ideal and anti-ideal. What distinguishes this method is the use of the measure of relative distance to the best solution, representing the pattern (ideal) and the worst solution, representing the anti-pattern (anti-ideal).

Stages of the research procedure in the TOPSIS method are as follows:

- to determine the decision matrix, according to Eq (2).
- to determine the normalized matrix, according to Eq (3).
- to determine the weighted normalized decision matrix:

$$x_{ij}^{*}=x_{ij}\times w_{i}$$ (8)

where: *w*_*i*_ is the weight of the *i*-th criterion

- to determine the ideal solution *S*^*+*^ and the non-ideal solution *S*^*-*^:

$$S^{+}=(x_{1}^{+},x_{2}^{+},x_{3}^{+},\ldots .x_{n}^{+})=\{\left(m{ax}_{i}x_{ij}|j\in B|),({min}_{i},x_{ij}|j\in C|\right)\}$$ (9)

$$S^{-}=(x_{1}^{-},x_{2}^{-},x_{3}^{-},\ldots .x_{n}^{-})=\{\left(m{in}_{i}x_{ij}|j\in B|),({max}_{i},x_{ij}|j\in C|\right)\}$$ (10)

- to determine the Euclidean distance of the object from the ideal variant *S*^*+*^ and the non-ideal variant *S*^*-*^:

$$d_{i}^{+}=\sqrt{\sum_{j=1}^{n}{\left(x_{ij}-x_{j}^{+}\right)}^{2}}$$ (11)

$$d_{i}^{-}=\sqrt{\sum_{j=1}^{n}{\left(x_{ij}-x_{j}^{-}\right)}^{2}}$$ (12)

- to determine the coefficient of relative closeness of the decision variants *S*_*i*_ to the ideal solution *S*^*+*^ (14):

$$P_{i}=\frac{d_{i}^{-}}{{d_{i}^{+}+d}_{i}^{-}}$$ (13)

The values of the TOPSIS synthetic measure belong to the range <0.1>, yet the higher the value reached by the synthetic measure, the higher position the object achieves in the ranking. The synthetic index (*P*_*i*_) calculated for each country orders the units linearly and allows the classification from the highest level to the lowest level.

#### 3.3.3 The MOORA method

The MOORA method by Brauers and Zavadskas [73] method belongs to the family of multi-criteria decision optimization methods. The application of this method makes it possible to select the best alternative, evaluated in terms of favorable and/or unfavorable criteria [74].

The main steps in applying the MOORA method are as follows:

- to create a decision matrix X with *m* number of alternatives and *n* number of criteria according to Eq (2).
- to create the normalized decision matrix according to Eq (3).
- to calculate the normalized score value for each alternative, taking into account all alternatives. In fact, the final score of each alternative is obtained by means of the following equation:

$$y_{i}^{*}=\sum_{j=1}^{t}x_{ij}^{*}-\sum_{j=t+1}^{n}x_{ij}^{*}\;\forall i$$ (14)

In this equation, $y_{i}^{*}$ represents the MOORA score for the i-th alternative. *j = 1*, *2*, *3……t and j = t+1*, *t+2……n* refer to the objectives that must be appropriately maximized (favorable criteria) and minimized (unfavorable criteria).

- If weights are included in the evaluations of alternatives with respect to specific criteria, then the MOORA score for each alternative is measured by the following equation:

$$y_{i}^{*}=\sum_{j=1}^{t}{w_{i}x}_{ij}^{*}-\sum_{j=t+1}^{n}{w_{i}x}_{ij}^{*}$$ (15)

where: *w*_*i*_ is the weight of the *i*-th criterion.

In the final ranking, alternatives with higher scores are desired. In other words, the alternative with the highest score is considered the best option and the alternative with the lowest score is seen as the worst option.

### 3.4. Method for determining the digital maturity level

All of the discussed methods allow solution ranking, from the best to the worst. However, none of them enable the determination of the level of digital maturity. Therefore, the authors decided to determine the level of digital maturity by using the mean value and standard deviation. The level classes of digital readiness in the MOORA and TOPSIS methods are defined as follows:

Class I—high level of digital maturity:

$$\begin{array}{c} P_{i}\geq \bar{P_{i}}+s_{P_{i}}\;(TOPSIS\;method)\\ y_{i}^{*}\geq \bar{y_{i}^{*}}+s_{y_{i}^{*}}\;(MOORA\;method) \end{array}$$ (16)

Class II–average high level of digital maturity:

$$\begin{array}{c} \bar{P_{i}}+s_{P_{i}}>P_{i}\geq \bar{P_{i}}\;(TOPSIS\;method)\\ \bar{y_{i}^{*}}+s_{y_{i}^{*}}>y_{i}^{*}\geq \bar{y_{i}^{*}}\;(MOORA\;method) \end{array}$$ (17)

Class III–average low level of digital maturity:

$$\begin{array}{c} \bar{P_{i}}>P_{i}\geq \bar{P_{i}}-s_{P_{i}}\;(TOPSIS\;method)\\ \bar{y_{i}^{*}}>y_{i}^{*}\geq \bar{y_{i}^{*}}-s_{y_{i}^{*}}\;(MOORA\;method) \end{array}$$ (18)

Class IV–low level of digital maturity:

$$\begin{array}{c} P_{i}<\bar{P_{i}}-s_{P_{i}}\;(TOPSIS\;method)\\ y_{i}^{*}<\bar{y_{i}^{*}}-s_{y_{i}^{*}}\;(MOORA\;method) \end{array}$$ (19)

On the other hand, in the VIKOR method, where the ideal solution is closest to or equals 0 the above ranges must be reversed, i.e., Eq (16), which determines the low level in the TOPIS and MOORA methods, allows for the high level to be determined in the VIKOR method, and so on:

Class I—high level of digital maturity:

$$Q_{j}<\bar{Q_{j}}-s_{Q_{j}}$$ (20)

Class II–average high level of digital maturity:

$$\bar{Q_{j}}>P_{i}\geq \bar{P_{i}}-s_{Q_{j}}$$ (21)

Class III–average low level of digital maturity:

$$\bar{Q_{j}}+s_{Q_{j}}>Q_{j}\geq \bar{Q_{j}}$$ (22)

Class IV–low level of digital maturity:

$$Q_{j}\geq \bar{Q_{j}}+s_{Q_{j}}$$ (23)

where: $\bar{P_{i}},\;\bar{Q_{j}}$ and $\bar{y_{i}^{*}}$ are the average values of *P*_*i*_, *Q*_*j*_ and $y_{i}^{*};\;s_{P_{i}},\;s_{Q_{j}}$ and $s_{y_{i}^{*}}$ are the standard deviation of *P*_*i*,_ *Q*_*j*_ and $y_{i}^{*}$.

### 3.5. The Shannon’s entropy method–to determine weights of determinants

The Shannon’s entropy method was used to determine the weights of the assumed determinants. The algorithm for determining the weights in this method is as follows:

- to construct the decision matrix according to Eq (2).
- to construct the normalized decision matrix:

$$x_{ij}=\frac{x_{ij}}{\sum_{i=1}^{m}x_{ij}}$$ (24)

- to determine entropy:

$$E_{j}=-k\sum_{t=1}^{m}x_{ij}ln(n_{ij})$$ (25)

where:

$$k=-\frac{1}{ln(n)}$$ (26)

where: *n*_*ij*_ is the proportion of samples in time *t* in the *i* indicator.

- to determine the variation level of entropy for each criterion (the degree of intrinsic divergence of scores from subsequent criteria) from Eq (27):

$$d_{j}=1-e_{j}$$ (27)

- to determine the weights (degree of importance) of the criteria according to Eq (28):

$$w_{i}=\frac{1-E_{j}}{\sum_{j=1}^{n}\left(1-E_{j}\right)}$$ (28)

### 3.6.Multidimensional scaling

In order to graphically represent the structure of similarity (or dissimilarity) between the studied objects based on the selected set of variables (features), multidimensional scaling (MDS) was used. The graphical presentation of results takes the form of a scatter plot of objects on a multidimensional scaling map. The smaller the distance between the analyzed objects, the more similar they are to one another. The graphical representation of results takes the form of a 2- or 3-dimensional map [75].

What distinguishes MDS from other similar techniques (e.g., factor analysis, cluster analysis) is that in the MDS method, there are no biases as to which factors may influence which dimensions [76].

Under the assumption that there are *n* points, denoted as *x1*, *x2*, *⋅⋅⋅⋅*, *xn*, the vector form is expressed as:

$$X={\left(x_{1},x_{2},{x_{3}}_{,}\ldots .x_{n}\right)}^{T}$$ (29)

then the algorithm for the multidimensional scaling process consists of the following steps:

- to determine the distance or dissimilarity between the number of *n* points using a certain algorithm, and then to obtain the inner product matrix B_*n*_ × n according to equation:

$$B_{ij}=\frac{1}{2}(-d_{ij}^{2}+\frac{1}{n}\sum_{j=1}^{n}d_{ij}^{2}+\frac{1}{n}\sum_{i=1}^{n}d_{ij}^{2}-\frac{1}{n^{2}}\sum_{i=1}^{n}\sum_{j=1}^{n}d_{ij}^{2})$$ (30)

- to calculate the eigenvalues *r*_*1*_, *r*_*2*_, *⋅⋅⋅ r*_*s*_ and eigenvectors *v* of the *B*_*n*_ *× n* dimensional matrix, which makes it possible to obtain the coordinates of all points in the dimensional matrix. In this way, a perceptual map can be created.

The quality of matching the output (reconstructed data) to the input data is measured by the so-called STRESS function. The smaller the value of this function, the better the match of the reconstructed distance matrix to the observed distance matrix. The STRESS function is defined as the root of the standardized sum of squares of the residuals between the input distances and the distances reconstructed by multidimensional scaling. It takes the following form [77]:

$$Stress=\sqrt{\frac{\sum_{i}\sum_{j}{\left(d_{ij}-\hat{d_{ij}}\right)}^{2}}{\sum_{i}\sum_{j}d_{ij}^{2}}}$$ (31)

where: *d*_*ij*_ is the distance or dissimilarity between points i; $\hat{d_{ij}}$ is a function on the input data, and it depends on whether one is dealing with metric or non-metric multidimensional scaling.

In metric multidimensional scaling, $d_{ij}=\hat{d_{ij}}$, while in non-metric scaling, the function is a monotonic transformation of the observed input data.

The criteria for evaluating the matching results are shown in Table 6.

**Table 6:** STRESS evaluation criteria [own elaboration based on [75]].

Value	Level
0	Excellent
0–0.25	Perfect
0.025–0.05	Good
0.05–0.10	Acceptable
0.10–0.20	Bad

Multidimensional scaling uses the Euclidean distance as a measure of distance between points. By denoting the coordinates of objects in dimension *r* (*r-dimension)* as *X*_*i*_ *= (X*_*i1*_, *X*_*i2*_,…….*X*_*ir*_), the Euclidean distance between *X*_*i*_ and *X*_*j*_ can be defined as:

$$d_{ij}=\sqrt{{\left(X_{i1}-X_{j1}\right)}^{2}+{\left(X_{i2}-X_{j2}\right)}^{2}+\cdots +{\left(X_{ir}-X_{jr}\right)}^{2}}$$ (32)

## 4. Results

The conducted research was divided into two stages: preliminary and fundamental. The preliminary analysis allowed the authors to determine statistical parameters of the indicators adopted for the study (subsection 4.1). The fundamental analysis, on the other hand, made it possible to both rank and assess the level of digital maturity among all and manufacturing enterprises in the CEE countries to later compare their similarities.

### 4.1. The statistical analysis of the determinants of digital maturity among enterprises in the CEE countries, including manufacturing enterprises

The indicators used for the study, characterizing the technological and social dimensions of digital maturity of enterprises in the CEE countries, were pre-processed and their basic statistical parameters were determined, which is summarized in Tables 7 and 8. These indicators were delineated for all and manufacturing enterprises (according to NACE rev. 2 activity).

**Table 7:** Selected statistical parameters of the indicators determining the level of digital maturity among all enterprises in the CEE countries.

Determinants	Mean	Median	Min	Max	Variance	Standard deviation	Coefficient of variation, %	Skewness	Kurtosis
Enterprises which have ERP software package	24.08	21.50	16.00	36.00	47.72	6.91	28.68	0.67	-1.21
Enterprises using software solutions like CRM	26.50	16.50	10.00	62.00	339.55	18.43	69.53	1.13	-0.11
Enterprises sending eInvoices	2.75	2.50	1.00	5.00	1.48	1.22	44.20	0.93	0.46
Analysis of big data from smart devices or sensors	3.92	4.00	2.00	7.00	1.90	1.38	35.21	0.68	1.26
Analysis of big data from the geolocation of portable devices	29.42	27.50	11.00	56.00	144.27	12.01	40.83	0.71	1.05
Purchase of cloud computing services used over the Internet	18.75	16.50	5.00	46.00	103.11	10.15	54.16	1.77	4.76
Purchase of high CC services	16.42	17.00	7.00	24.00	17.72	4.21	25.64	-0.63	1.89
Use of interconnected devices or systems that can be monitored or remotely controlled via the Internet	5.67	5.50	2.00	12.00	6.97	2.64	46.59	1.07	2.21
Use of smart meters, smart lamps, smart thermostats to optimise energy consumption in the enterprise’s premises	4.83	5.00	0.00	8.00	4.88	2.21	45.70	-0.72	0.72
Use of sensors or RFID tags to monitor or automate production processes	1.67	1.50	1.00	3.00	0.61	0.78	46.71	0.72	-0.79
Analysis of big data internally using machine learning	3.58	3.00	2.00	6.00	1.90	1.38	38.48	0.42	-1.18
Use of 3D printing	5.67	6.50	3.00	8.00	3.15	1.78	31.33	-0.45	-1.46
Use of industrial or service robots	17.08	17.00	6.00	26.00	38.45	6.20	36.30	-0.45	-0.08
Enterprises that provide training to develop/upgrade ICT skills of their personnel	24.08	21.50	16.00	36.00	47.72	6.91	28.68	0.67	-1.21

**Table 8:** Selected statistical parameters of the indicators determining the level of digital maturity among manufacturing enterprises in the CEE countries.

Determinants	Mean	Median	Min	Max	Variance	Standard deviation	Coefficient of variation, %	Skewness	Kurtosis
Enterprises which have ERP software package	33.55	32.00	20.00	52.00	111.87	10.58	31.53	0.51	-0.76
Enterprises using software solutions like CRM	19.27	17.00	11.00	37.00	67.42	8.21	42.60	1.26	1.02
Enterprises sending eInvoices	24.55	18.00	8.00	54.00	238.27	15.44	62.89	1.23	0.24
Analysis of big data from smart devices or sensors	2.91	3.00	1.00	4.00	0.89	0.94	32.45	-0.66	0.20
Analysis of big data from the geolocation of portable devices	2.82	3.00	1.00	4.00	0.76	0.87	31.01	-0.69	0.78
Purchase of cloud computing services used over the Internet	27.45	24.00	9.00	55.00	160.07	12.65	46.08	0.91	1.16
Purchase of high CC services	16.18	13.00	4.00	44.00	107.96	10.39	64.21	2.11	5.66
Use of interconnected devices or systems that can be monitored or remotely controlled via the Internet	18.09	17.00	5.00	46.00	110.09	10.49	58.00	1.99	5.55
Use of smart meters, smart lamps, smart thermostats to optimise energy consumption in the enterprise’s premises	7.27	8.00	2.00	16.00	14.22	3.77	51.85	0.94	2.22
Use of sensors or RFID tags to monitor or automate production processes	6.55	5.00	1.00	27.00	50.07	7.08	108.11	2.84	8.74
Analysis of big data internally using machine learning	1.00	1.00	0.00	3.00	0.80	0.89	89.44	1.02	1.56
Use of 3D printing	7.45	7.00	3.00	13.00	9.87	3.14	42.15	0.57	-0.55
Use of industrial or service robots	14.27	14.00	7.00	23.00	24.02	4.90	34.34	0.29	-0.63
Enterprises that provide training to develop/upgrade ICT skills of their personnel	15.82	16.00	5.00	28.00	49.96	7.07	44.69	0.02	0.00

Based on the results, it can be concluded that the determinants of digital maturity selected for analysis (treated as variables), meet the condition of diagnostic features, which must be marked with significant variation (above 10%). For these determinants, the value of the coefficient of variation was found to be characterized by significant spread. The highest value of the coefficient of variation for manufacturing enterprises was found for the determinants "use of sensors or RFID tags to monitor or automate production processes (to manage logistics, to track the movement of products)"– 108.11% and for "analysis of big data internally using machine learning"– 89.44%. For all enterprises, the highest value of the coefficient of variation within the studied group was reported for the determinant "enterprises using software solutions like Customer Relationship Management (CRM)"– 69,53%. The lowest value of the coefficient of variation for all enterprises was found for the determinant “purchase of high CC services (25.64%), and for manufacturing enterprises, it was “analysis of big data from the geolocation of portable devices” (31.01%).

The difference between the median and mean values of the determinants shows distribution asymmetry. The positive sign (median greater than the mean value) characterizes the left-sided asymmetry (left-skewed distributions), indicating the predominance of countries with high determinant values. The negative sign is associated with the right-sided asymmetry (right-skewed distributions), indicating the predominance of countries with low determinant values.

The left-skewed asymmetric distributions in the case of all enterprises were found for the determinants “analysis of big data from smart devices or sensors”, “purchase of high CC services, use of smart meters, smart lamps, smart thermostats to optimize energy consumption in the enterprise’s premises”, and “use of 3D printing”.

In the case of manufacturing enterprises, the left-skewed asymmetric distributions were found for the determinants “analysis of big data from smart devices or sensors”, “analysis of big data from the geolocation of portable devices”, “use of smart meters, smart lamps, smart thermostats to optimize energy consumption in the enterprise’s premises”, and “enterprises that provide training to develop/upgrade ICT skills of their personnel”. For the remaining determinants of all and manufacturing enterprises, the determinants of digital maturity adopted the right-skewed asymmetric distributions.

Since all determinants are stimulants, it is reasonable to assume that only for determinants with the left-skewed asymmetry distributions, a favorable situation in terms of their level was noted. The predominance of countries with high values was observed.

**Fig 3:**
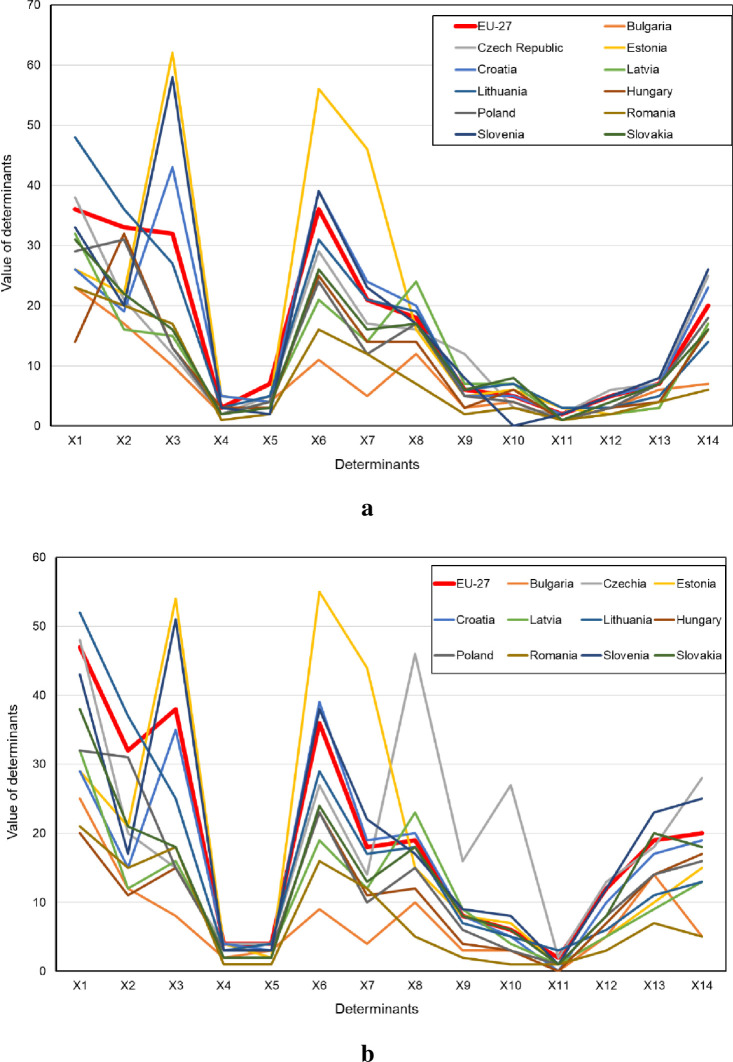
Summary of the values of studied determinants with regard to digital maturity among enterprises in the CEE countries compared to the EU countries (a–all enterprises, b–manufacturing enterprises).

With regard to the comparison of the CEE countries to the entire European Union (EU-27), Fig 3 presents the distribution of values of the digitization determinants for enterprises in these countries.

The results clearly show that the digital maturity determinants in the CEE countries are low versus the average values across the EU. The highest results among the CEE countries were obtained by the Czech Republic, Slovenia, Estonia, Croatia, and the lowest results by Bulgaria and Romania.

### 4.2. The assessment and ranking of digital maturity among enterprises in the CEE countries

The use of the presented MCDM methods (TOPSIS, MOORA and VIKOR) allowed for the ranking of studied CEE countries in terms of the level of digital maturity among their enterprises. All diagnostic variables adopted for the study were stimulants.

**Fig 4:**
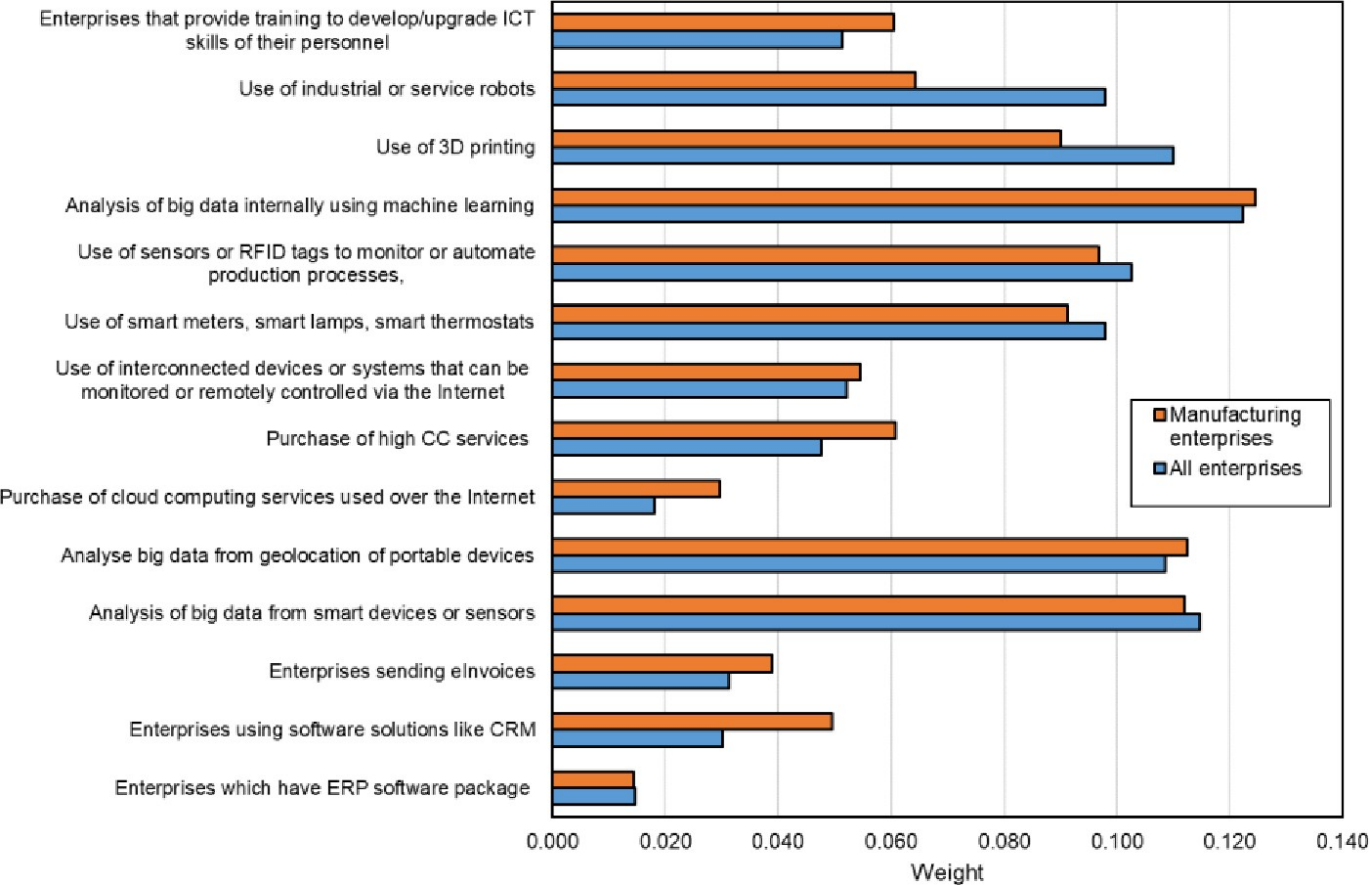
Values of weights for the determinants of digital maturity for all and manufacturing enterprises in the CEE countries.

The values of weights for these determinants, calculated by the entropy method (Eqs 24–28), are presented in Fig 4, while the total values of weights for each dimension are summarized in Table 9.

**Table 9:** Total values of weights for individual dimensions of digital maturity of enterprises.

Area	All enterprises	Manufacturing enterprises
Big data analytics	0.223	0.224
Artificial intelligence	0.122	0.125
Cloud computing	0.066	0.090
3D printing	0.110	0.090
Robotics	0.098	0.064
Integration of internal processes	0.045	0.064
Integration with customers/suppliers, supply chain management	0.031	0.039
Internet of Things	0.253	0.243
Digital skills	0.051	0.060

The weights calculated for individual dimensions of digital maturity indicate that the dimensions Internet of things, big data analytics and artificial intelligence are the most important criteria for assessing the digital maturity of enterprises in the CEE countries.

The obtained values demonstrate that the highest weights should be adopted for the determinant “analysis of big data internally using machine learning” (artificial intelligence dimension) and “analysis of big data from smart devices or sensors” (big data dimension), and the lowest weights for “enterprises which have ERP software package to share information between different functional areas” (integration of internal processes dimension) and “purchase of cloud computing services used over the Internet” (cloud computing dimension). The weights determined by the entropy method for individual determinants of digital maturity define the degree of their disorder as a set of data, namely the degree of its uniqueness within the examined set of countries.

**Fig 5:**
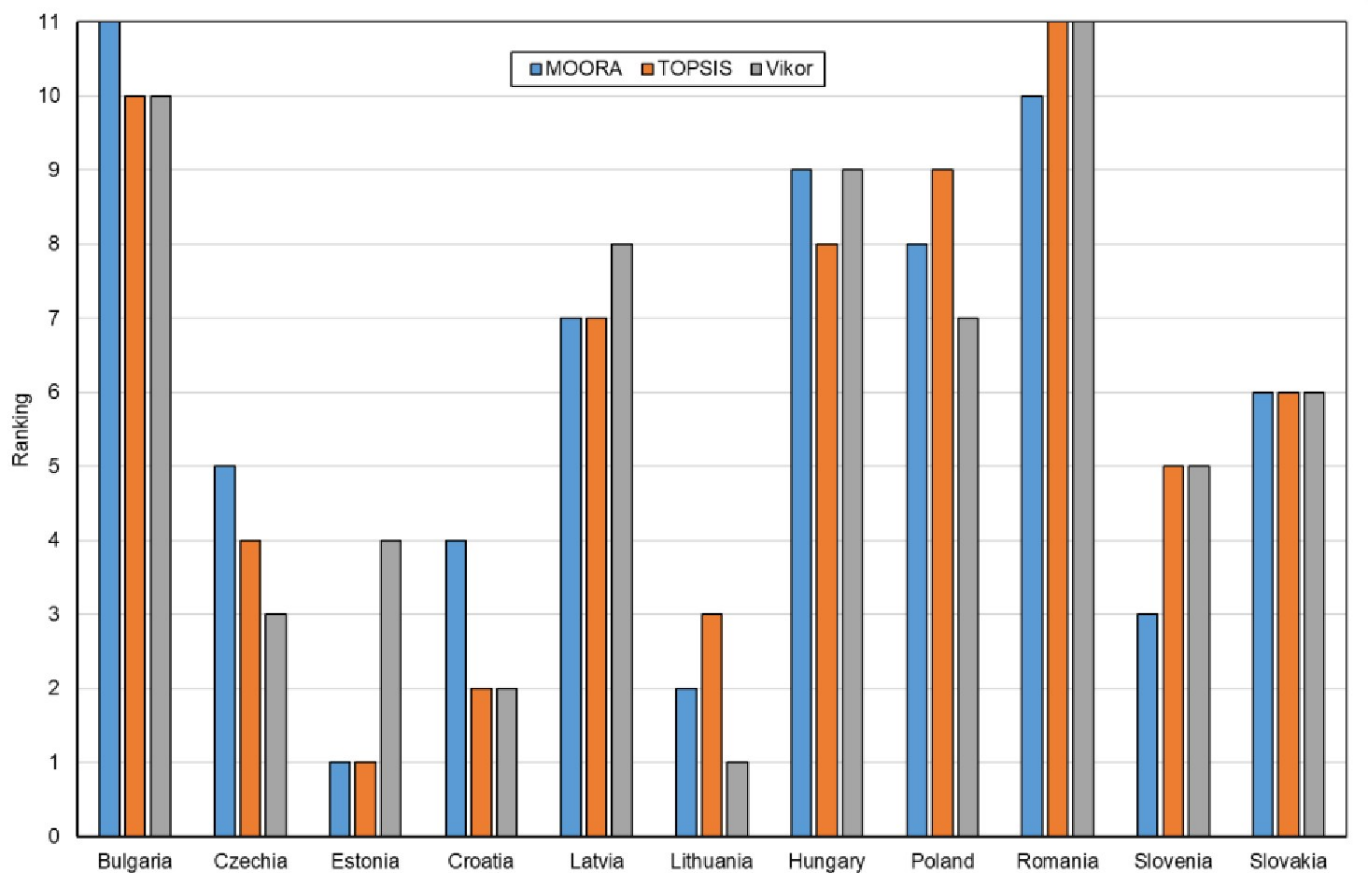
Classification (ranking) of the CEE countries in terms of digital maturity among all enterprises using the MOORA, TOPSIS and VIKOR methods.

**Fig 6:**
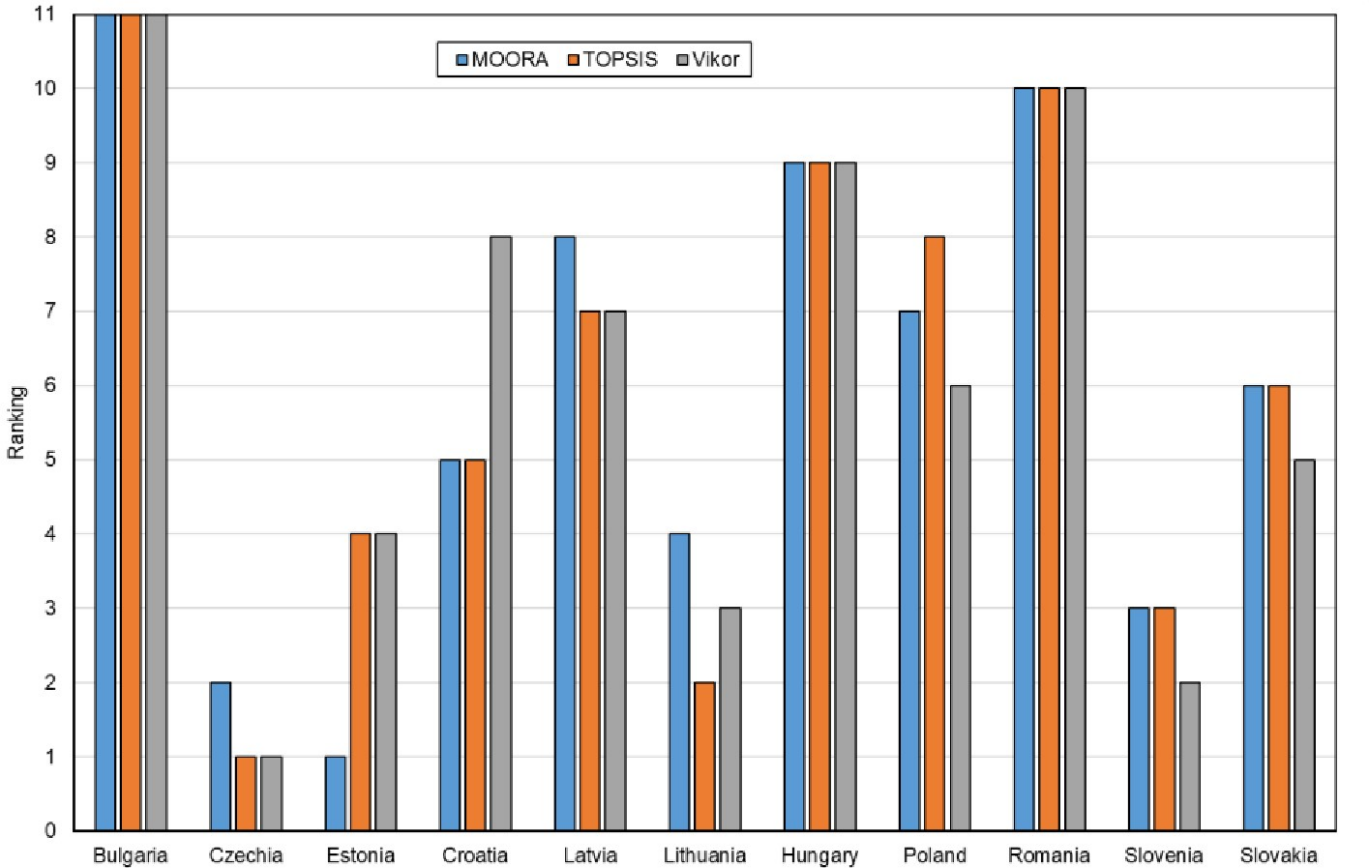
Classification (ranking) of the CEE countries in terms of digital maturity among manufacturing companies using the MOORA, TOPSIS and VIKOR methods.

In the next stage of the analysis, ordering indices were determined for each method, i.e.: the $y_{i}^{*}$ index in the MOORA method, the *Pi* index in the TOPSIS method and the *Qi* index in the VIKOR method. The results are summarized in Table 10. Based on the values of these indices, a classification (ranking of countries) was made in terms of the level of digital maturity in all and manufacturing enterprises in the CEE countries belonging to the EU. The results of this classification are shown in Figs 5 and 6.

**Table 10:** Values of indices classifying the digital maturity among all and manufacturing enterprises by the MOORA, TOPSIS and VIKOR methods for the CEE countries.

Countries	All enterprises						Manufacturing enterprises					
	MOORA		TOPSIS		Vikor		MOORA		TOPSIS		Vikor	
	$y_{i}^{*}$	Ranking	*Pi*	Ranking	*Qi*	R	$y_{i}^{*}$	Ranking	*Pi*	Ranking	*Qi*	Ranking
**Bulgaria**	0.014	11	0.124	10	0.850	10	0.010	11	0.040	11	0.926	11
**Czechia**	0.024	5	0.589	4	0.189	3	0.029	2	0.887	1	0.000	1
**Estonia**	0.038	1	0.778	1	0.405	4	0.032	1	0.337	4	0.464	4
**Croatia**	0.029	4	0.683	2	0.023	2	0.023	5	0.206	5	0.725	8
**Latvia**	0.022	7	0.344	7	0.729	8	0.018	8	0.188	7	0.565	7
**Lithuania**	0.031	2	0.679	3	0.009	1	0.026	4	0.556	2	0.405	3
**Hungary**	0.019	9	0.265	8	0.772	9	0.014	9	0.084	9	0.846	9
**Poland**	0.021	8	0.251	9	0.695	7	0.019	7	0.183	8	0.550	6
**Romania**	0.016	10	0.033	11	1.000	11	0.012	10	0.045	10	0.923	10
**Slovenia**	0.031	2	0.465	5	0.478	5	0.028	3	0.387	3	0.392	2
**Slovakia**	0.022	6	0.386	6	0.682	6	0.020	6	0.194	6	0.528	5

**Fig 7:**
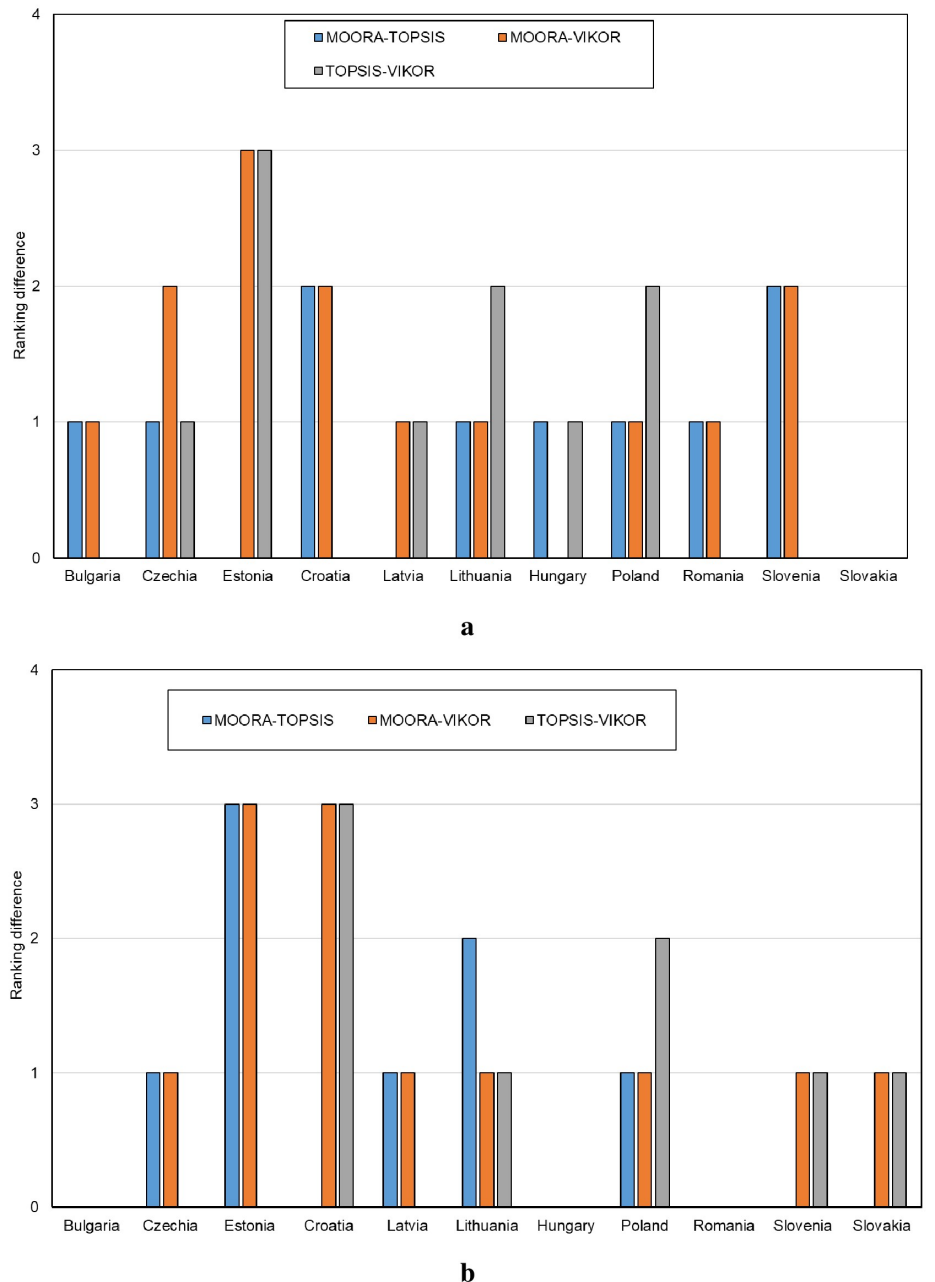
Absolute differences in the rankings between the methods MOORA-TOPSIS, MOORA-VIKOR, TOPSIS-VIKOR (a–all enterprises, b–manufacturing enterprises).

The results of the classification (ranking) of the CEE countries show that for some countries (alternatives) the findings are the same or very similar, and for others–different. For example, Slovakia in terms of digital maturity among all enterprises in the ranking for all methods was found to be on the 6^th^ place, while the Czech Republic—using the MOORA method–on the 5^th^ place, using the TOPSIS method–on the 4^th^ place, and using the VIKOR method–on the 3^rd^ place. The differences between the rankings for individual methods for all enterprises are shown in Fig 7A and for manufacturing enterprises in Fig 7B.

The analysis of differences showed that complete correspondence for the ranking of digital maturity among enterprises between the applied methods was observed for Slovakia, and for manufacturing enterprises–for Romania and Hungary. In general, the magnitude of differences in the rankings was found to be insignificant (difference in one or two positions). The difference in 3 positions in the ranking was reported for Estonia (Fig 7) and Croatia, yet only for manufacturing companies (Fig 7B).

**Fig 8:**
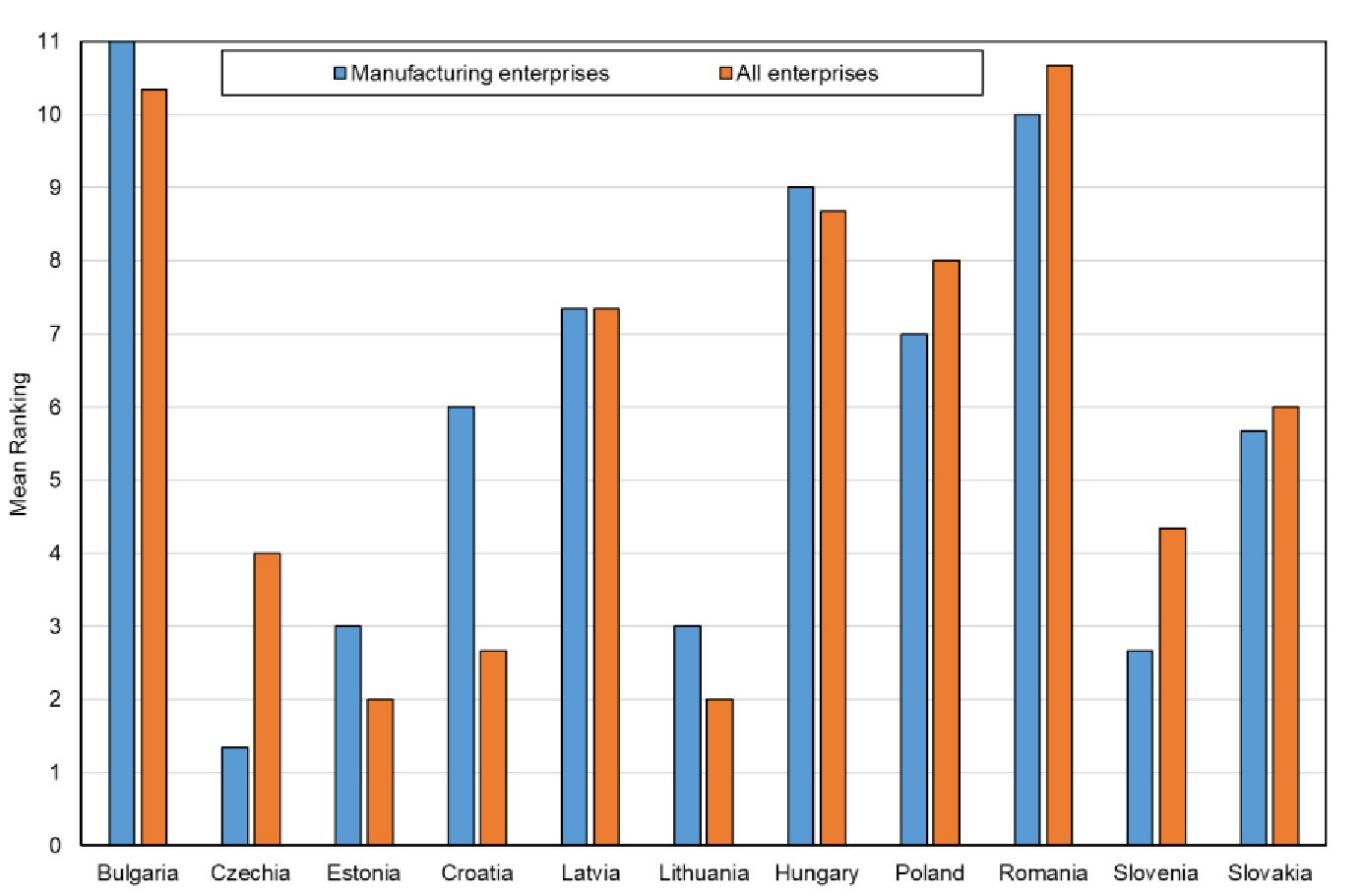
The ranking of the CEE countries in terms of digital maturity among their enterprises by the mean-rank method.

The results of the ranking of the CEE countries in terms of the digital maturity among all and manufacturing enterprises showed similar results for most of the countries, and only for several were they different. In order to obtain a single reliable ranking of studied countries, the mean-rank method was utilized to combine the results obtained using the MOORA, TOPSIS and VIKOR methods. The mean-rank method seems to be, in this case, most simple and objective, as it can provide an unambiguous result [78,79]. The ranking for 11 countries in terms of digital maturity among all and manufacturing enterprises is shown in Fig 8.

Thus, the final ranking of the CEE countries in terms of digital maturity is as follows:

- for all enterprises: Estonia > Lithuania > Croatia > Czechia > Slovenia > Slovakia > Latvia > Poland > Hungary > Bulgaria > Romania,
- for manufacturing companies: Czechia > Slovenia > Estonia > Lithuania > Slovakia > Croatia > Poland > Latvia > Hungary > Romania > Bulgaria.

The results showed that in terms of the level of digital maturity among all enterprises of the CEE countries, Estonia and Lithuania were found to perform best, and Bulgaria and Romania were found to perform worst. In addition, Bulgaria and Romania were also observed to have the worst results in terms of the level of digital maturity among manufacturing enterprises. Within this group of companies, the best performers were the Czech Republic, Slovenia and Estonia.

**Fig 9:**
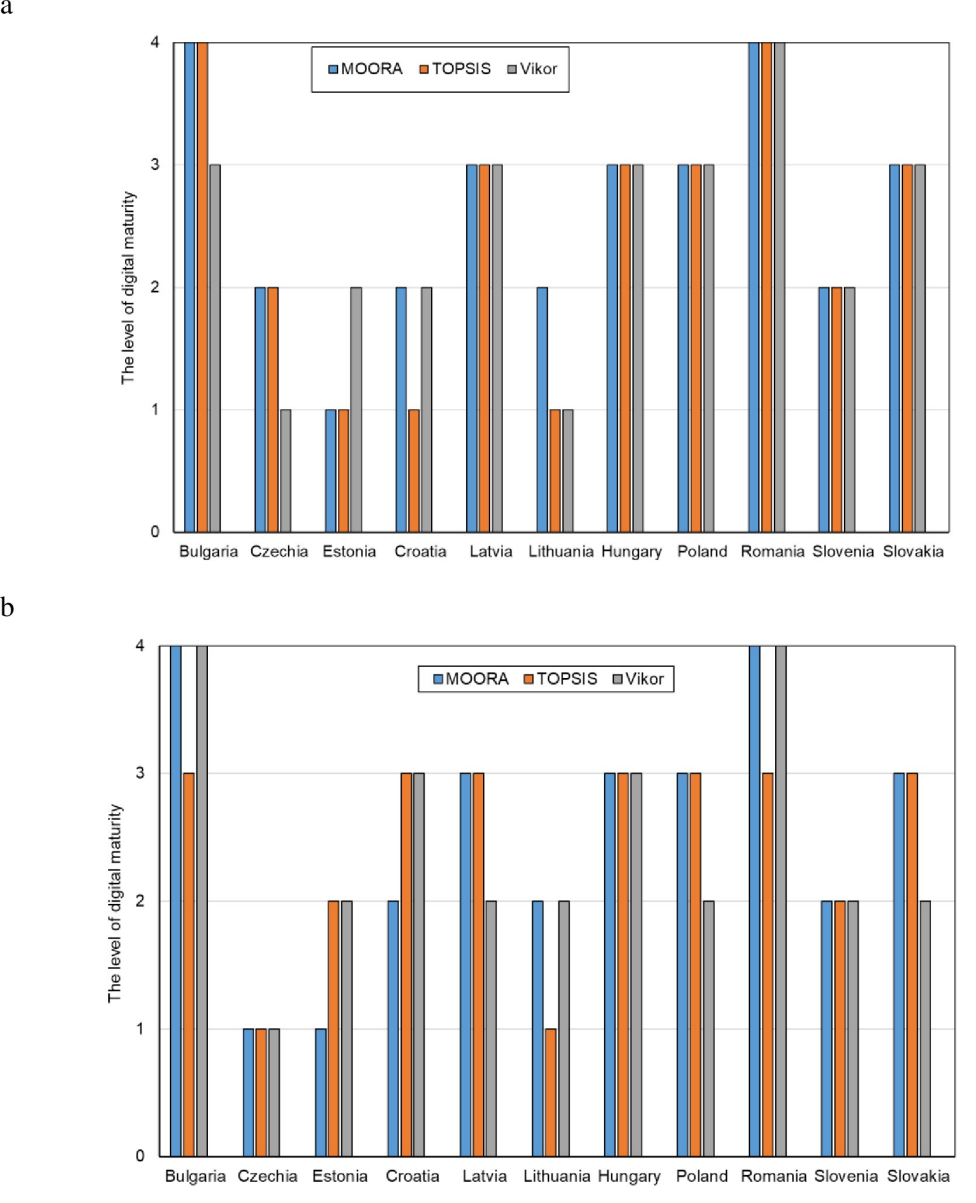
Assessment of the level of digital maturity among enterprises in the CEE countries (a–all enterprises, b–manufacturing enterprises) (1 –high level, 2 –average high level, 3 –average low level, 4 –low level).

**Fig 10:**
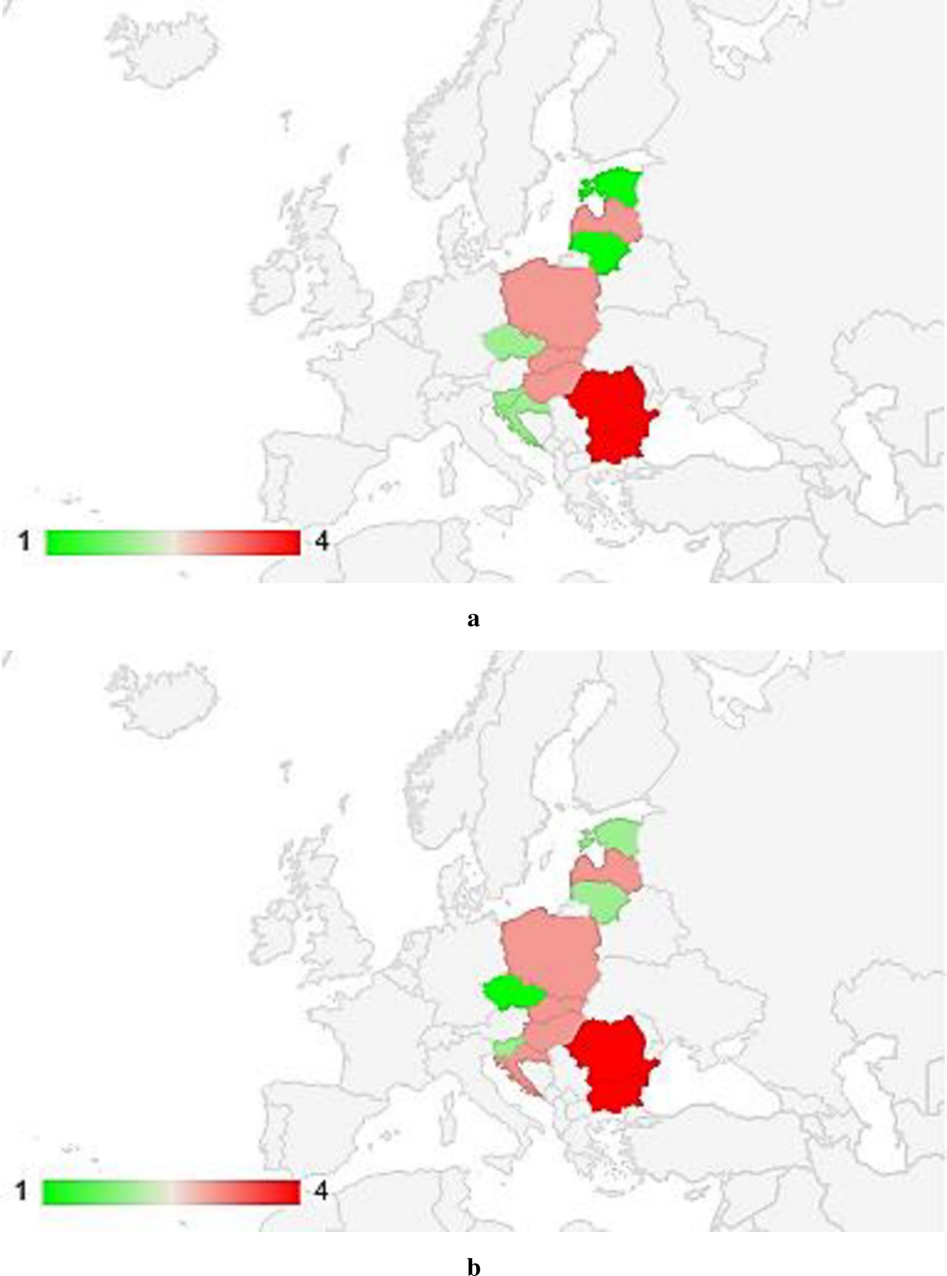
Assessment of the level of digital maturity among enterprises in the CEE countries by the mean- rank method (1 –high level, 2 –average high level, 3 –average low level, 4 –low level).

Based on the mean values and standard deviation (12–15), the digital maturity classes of the CEE countries were also determined. These classes for individual countries were determined for each method separately (Fig 9), and then using the mean-rank method, the levels were evaluated (Fig 10).

Countries that were found to have a high level of digital maturity (according to the mean-rank method), for all enterprises (over 10 employees, excluding the financial sector), included Estonia and Lithuania. However, when analyzing the level of digital readiness of enterprises only from the manufacturing sector, it turned out that the Czech Republic was shown to have a high level.

An average high level of digital maturity among all enterprises was reported for the Czech Republic, Croatia and Slovenia, and among manufacturing enterprises for Estonia, Lithuania and Slovenia.

An average low level among all enterprises was found for Latvia, Slovakia, Poland and Hungary, and among manufacturing enterprises for Hungary, Poland, Slovakia, Croatia and Latvia. On the other hand, a low level of digital maturity among all and manufacturing enterprises was reported for Romania and Bulgaria.

For most CEE countries, the level of digital maturity among all and manufacturing enterprises was found to be the same. The exception was the Czech Republic, being the leader in terms of the level of digital maturity among manufacturing enterprises, and in the case of all enterprises, the level of this maturity was defined as average high. The opposite situation was reported for Estonia and Lithuania, being the leaders in the level of digital maturity for all enterprises, and for manufacturing enterprises, the level of digital maturity was assessed as average high.

**Fig 11:**
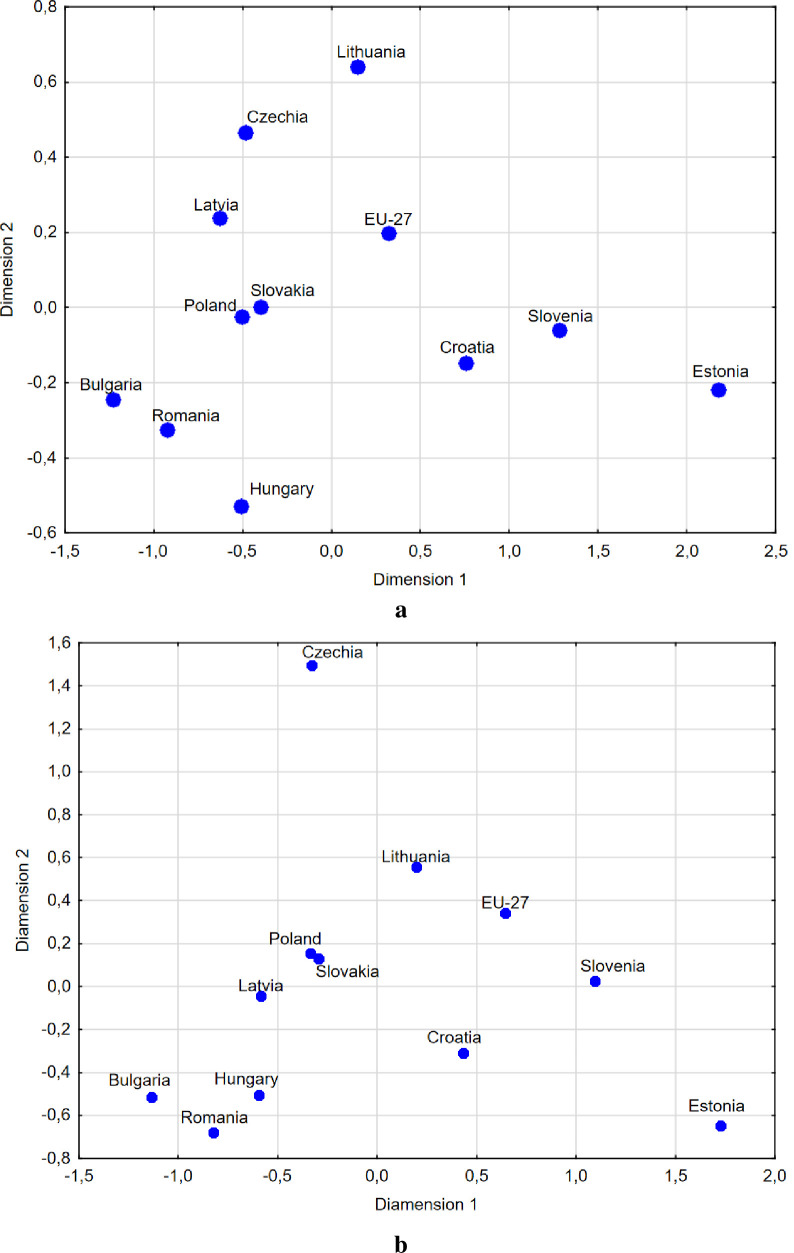
Similarities between the CEE countries in terms of digital maturity characterized by the set of 14 determinants in a two-dimensional space (a–all enterprises, b–manufacturing enterprises).

In order to illustrate similarities between the CEE countries in terms of digital maturity characterized by the set of 14 digitization determinants, using the multidimensional scaling method, scatter plots in a two-dimensional space were prepared (Fig 11). The quality of matching the output to the input data was measured using the STRESS function, the results of which are presented in Table 11. Also, these graphs include the value for all EU countries, which represents the average level of digital maturity among enterprises across the EU.

**Table 11:** The value of STRESS function.

Variants	Value of STRESS function
All enterprises	0.0412
Manufacturing enterprises	0.0542

The results of the analysis presented in Fig 11 indicate that the distances between the CEE countries in terms of digital maturity among all and manufacturing enterprises vary significantly. In general, the results show that they have little similarity in terms of digital maturity. However, in the case of digital maturity among manufacturing companies, a slightly greater narrowing of the borders between these countries can be seen, which means that the disparity between them in this regard is smaller. Changes in the distance between countries in terms of digital maturity among all enterprises versus manufacturing enterprises can be interpreted as changes in digital transformation occurring faster in manufacturing enterprises than in other sectors of the economy. In general, the closer countries are in a two-dimensional space to each other, the more similar they are (the more distant they are, the more dissimilar they are).

It can also be observed that Poland and Slovakia show high similarity in terms of digital maturity both among all and manufacturing enterprises. In terms of digital maturity among all enterprises, Croatia, Estonia, Slovakia, the Czech Republic and Lithuania were found to exceed the EU27 average for most determinants, while Estonia, Slovakia and the Czech Republic were found to exceed the EU27 average for manufacturing enterprises. The worst results in terms of digital maturity versus the EU average were reported for Hungary, Romania and Bulgaria.

## 5. Discussion

As a result of the conducted research, rankings were made and the level of digital maturity among all and manufacturing enterprises in the CEE countries was assessed. Three methods from the group of multi-criteria decision-making methods (MOORA, TOPSIS, VIKOR) were used for analysis. In addition, the use of multidimensional scaling allowed for the differentiation of these countries in terms of the level of digital maturity, characterized by the set of 14 selected determinants in a two-dimensional space. Central to the analysis was the selection of determinants that characterized the most important 9 areas related to the digitization of enterprises.

Thus, the analytical methods, together with the determinants, made it possible to assess the digital maturity of individual countries. For each of the methods used for the analysis, the order of studied countries in terms of the level of digital maturity was also determined. Additionally, by means of the mean-rank method, the results obtained were averaged to make the final classification of the CEE countries in terms of digital maturity.

It should be emphasized that such a broadly ranked assessment of digital maturity has never been conducted for the group of CEE countries. Also, the application of three different multi-criteria decision-making methods (MOORA, TOPSIS, VIKOR) and the method of multidimensional scaling is a new, original and informative approach to the study of digital maturity in the CEE countries.

When analyzing the results, it can be stated that in terms of the assessment of digital maturity among all enterprises (over 10 employees, excluding the financial sector) in the CEE countries, a high level was found in Estonia and Lithuania, and for enterprises in the manufacturing sector—in the Czech Republic. In these countries, the determinants of digital maturity were at a very high level, exceeding, in most cases, the average values for the EU27. One of the reasons for this state of affairs is the fact that these countries, as indicated by the research results presented in one paper [80], are characterized by a high level of innovation and competitiveness.

The Czech Republic, Croatia and Slovenia were found to have an average high level of digital maturity for all enterprises, and Estonia, Lithuania and Slovenia for manufacturing enterprises.

An average high level of digital maturity among all enterprises was shown for the Czech Republic, Croatia and Slovenia, and among manufacturing enterprises for Estonia, Lithuania and Slovenia.

An average low level of digital maturity among all enterprises was found for Latvia, Slovakia, Poland and Hungary, and among manufacturing enterprises for Hungary, Poland, Slovakia, Croatia, and Latvia. On the other hand, a low level of digital maturity among all and manufacturing enterprises was found for Romania and Bulgaria.

Both all and manufacturing enterprises operating in other countries were reported to be still insufficiently involved in processes related to robotization, the use of 3D printing, big data analytics, and the use of cloud computing. This could have a very negative impact on their competitiveness and financial status in the years to come.

Both Bulgaria and Romania are facing significant challenges in improving their level of digital maturity [81]. The level of digitalization in these countries, compared to other CEE countries, is relatively low. This is due to many factors. One of the reasons is low expenditure on R&D. In 2019 (the latest available data), Romania ranked lowest in this respect, as its R&D expenditure amounted to only 0.48% of GDP, while in Estonia, the country with the highest level of digital maturity among all enterprises, it was 1.61% [13].

**Fig 12:**
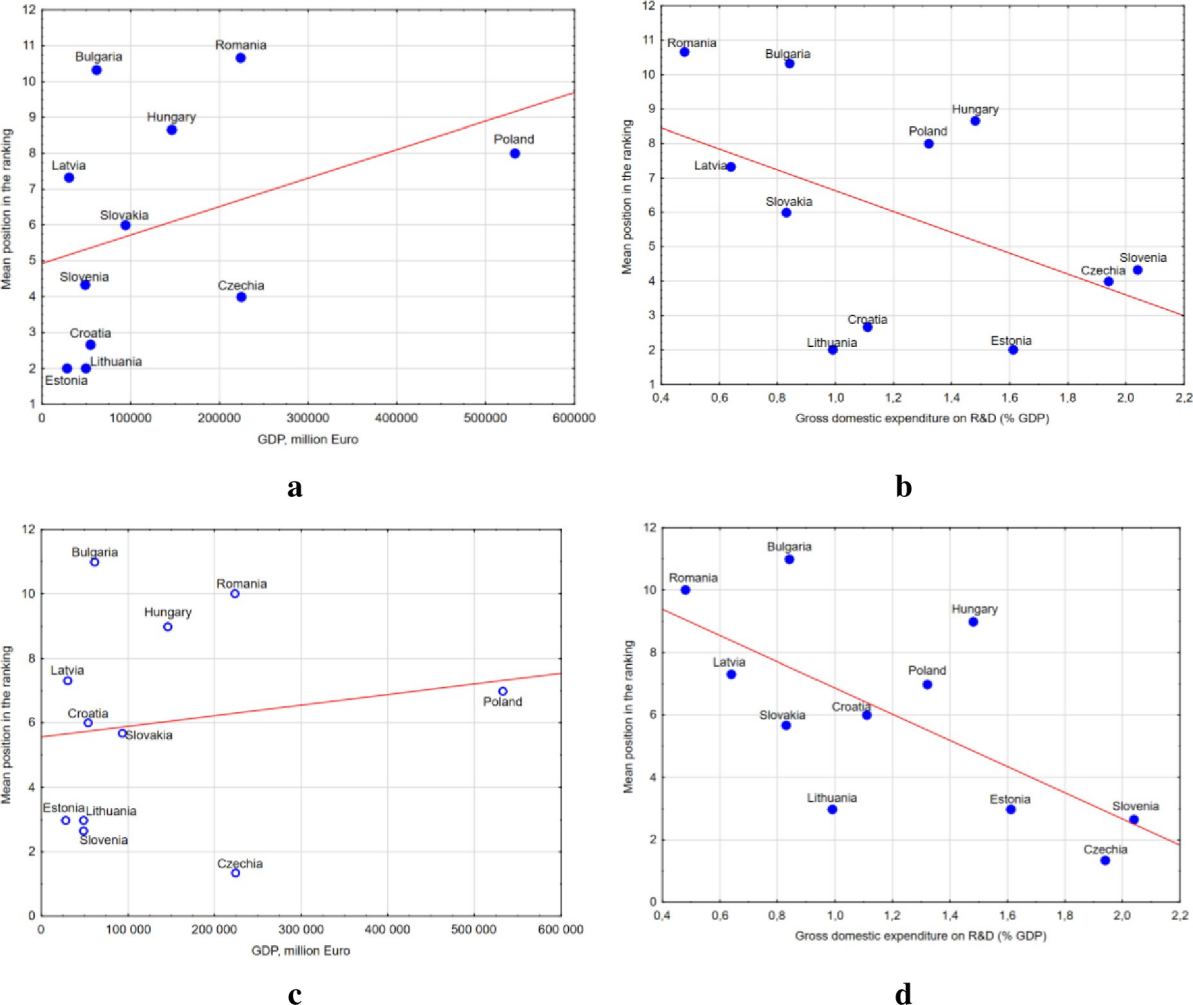
Ratio of GDP value and % GDP spent on R&D vs ranking position in terms of digital maturity (a-GDP value vs ranking position for all enterprises; b-% GDP spent on R&D vs ranking position for all enterprises; c- GDP value versus ranking position for manufacturing enterprises; d-% GDP spent on R&D vs ranking position for manufacturing enterprises).

It is worth noting that the level of digital maturity (ranking position) among companies in the CEE countries is less dependent on the value of the country’s GDP or % of GDP spent on R&D (Fig 12). The results indicate that countries with higher GDP values, or those spending more on R&D, often achieved lower positions in the ranking. For example, Poland, being the country with the highest GDP value among the CEE countries, ranked 8^th^ in the ranking of digital maturity (Fig 8). At the same time, the country ranked 5^th^ in terms of % of GDP spent on R&D. An equally unfavorable relationship was found for manufacturing enterprises (Fig 12C and 12D).

Estonia, Lithuania and Croatia ranked highest in terms of the ratio of GDP value to the position in the ranking of digital maturity among all enterprises, and in terms of the ratio of GDP value spent on R&D to the position in the ranking, it was Lithuania and Croatia.

Immensely large and, so far, underutilized potential for digitalization development opportunities exists in Hungary, which is the result of large investments made in the country by Germany, Japan and South Korea. Investments by foreign partners not only generate new jobs, but also contribute to the development of the R&D sector, which supports these investments in highly innovative technologies.

The Visegrad Group, consisting of Poland, the Czech Republic, Slovakia and Hungary, should also have a large and positive impact on the development and digital maturity of enterprises in the case of CEE countries [82]. This group is increasingly involved in the development of digitization in these countries. Joint projects create greater chances to obtain funding and implement the results of these undertakings.

The findings of this study show that the CEE countries, despite favorable conditions in the EU, are not fully using the opportunities created by this community in the area of digitization. In this regard, it seems reasonable to expand cooperation between these countries and implement larger joint ventures. As indicated by Novak et al. [80], the CEE market is worth around 1.4 trillion Euros, which ranks the region as the 12^th^ largest economy in the world. It is therefore huge potential that should be well used, especially in the process of digitization of the economy.

An important factor that should enable cooperation between the countries is the fact that they are distinguished by a high level of market openness, belong to the same cultural circle and have a similar history. Also, they are facing common challenges, especially in terms of stemming the outflow of skilled workers to other, wealthier countries and the need to retrain a significant portion of the workforce [83,84]. The retraining of workers will be a major challenge for, e.g., Poland, which is additionally forced to carry out a very thorough energy transition. In this respect, the digitalization of the economy should be used as an opportunity to create new jobs and modernize the country.

The results provide very large opportunities for interpretation and conclusions in terms of individual areas and determinants (indicators) adopted for the study. Their analysis showed a great diversity of individual areas of digitalization processes in the CEE countries and enterprises. Therefore, closer cooperation between these countries regarding digitization seems obvious. The joint development of the digital economy should bring positive results. At the same time, the coalition of these countries should have much more clout in creating financial policy across the EU.

After all, it is in the interest of the CEE countries, and the EU as a whole, to implement new technologies and build a digital economy as quickly as possible. Achieving higher values for the determinants of digitization among enterprises requires both cooperation and solidarity between countries. The values of these determinants in the coming years can be used as a basis to assess the changes introduced and the effectiveness of the EU policy on digitalization.

## 6. Limitations and futhure direction

The methodology, the research and the results allowed the authors to formulate an opinion on the limitations of this methodology and future research directions.

In terms of limitations that may have affected the results, it is necessary to mention the determinants of digital maturity that were used for the research. Although an effort was made to adopt them to reflect all the most relevant areas related to digital transformation, it is obvious that they do not fully describe the process. Literature analysis and practical experience indicate that this set could be further enriched, and the scope of the research could be broadened. However, this process would increase the already extensive research material included in the publication. Nevertheless, the use of more indicators for analysis may provide a direction for further research. From the point of view of further research, the selection of indicators (determinants) and the analysis of their variability over time for individual countries would also be important. In this context, it would also be interesting to conduct research on the level of digital maturity of individual sectors of the economy of the countries studied (e.g., transport, energy, agro-food industry, environmental protection, telecommunications, engineering, financial services, health care, etc.). This may concern both the assessment of the current state and changes in recent years, as well as forecasts for the future. Research on service companies, where the process of digital transformation is very dynamic, would also be immensely relevant. A certain limitation of the publication, but also a direction of future research, is the extension of the analysis to other countries (e.g., all European Union countries), as well as the choice of time horizon and methods applied.

Therefore, it can be concluded that the study concerns a selected part of the current and very interesting issues, which in the coming years, due to the importance and development of the processes associated with digital transformation, will be developed and undertaken by subsequent researchers.

## 7. Conclusion

The development of information technology and ICT sciences and the practical application of their achievements have caused permanent and increasingly dynamic changes in the global economy. Their effect is the process of digitalization, which now covers virtually all areas of life. On the one hand, it gives a chance for development, and on the other hand, it is connected with many threats. Global changes related to the process of digitization cause its effects to be felt both at global and local levels. This applies to individual countries and their groups as well as individual companies. Of particular importance are the processes associated with changes at the level of enterprises, which have a significant impact on local and national economies as well as on employees and consumers.

The importance of the digitization process is evidenced by the fact that the legislation of individual countries and groups of countries is quickly adapted to this situation. The significance of digital transformation is also recognized by the European Union. The digitalization of the economy is seen as an opportunity for the development of less wealthy countries and strengthening Europe’s position in the world, and above all, as a chance to improve the lives of its citizens. The Central and Eastern European countries are immensely important in the EU activities in the field of digitization. Their geo-political location and social and economic potential may determine the future of the entire EU, especially because the EU assumes very ambitious economic, social and climate plans for the coming years. Without the development of the CEE countries, it will be impossible to achieve these goals.

In this context, achieving success in the digitization process of the economies of these countries becomes one of the EU’s priorities.

Thus, this state of affairs fully justifies conducting research to assess the digital maturity of companies in these countries. The knowledge gained from the implementation of this research can form the basis for the development of effective policies for the implementation of digital and energy transformation, which are closely related to the climate policy.

The presented approach to the analysis of the problem of digital maturity in the CEE countries undoubtedly represents a new approach to this topic. This applies both to the advanced analytical tools used as well as to the determinants included in the analysis related to the key areas of the digitalization process. Until now, neither such a broad scope nor advanced analytical tools have been used to study digitization processes in these countries.

The results are immensely intriguing and allow for the assessment of digital maturity among these countries at many levels. First of all, they constitute a substantial database, only some of which is discussed in this paper. Their comprehensiveness and reference to many issues related to digitization makes them an important source of information on digitization in the CEE countries and the basis for research on other countries.

These results also showed the diversity of the EU countries, which on one hand constitutes their strength, but can also be the cause of many problems. As far as digitalization is concerned, it seems that this diversity, visible in the results of the conducted analyses, can be an opportunity for the dynamic development of the whole region. The relatively young and well-educated societies of the CEE countries, their high ambitions to improve their economic status, and also the low costs of labor mean that the potential of these countries to implement new technologies is very high. This entails that in the coming years, they can achieve these ambitious goals, also in the digitization process.

The presented results can be widely used to improve the funding process for the digitalization activities of the EU countries and to target this funding to the most neglected areas. The division of the CEE countries into similar classes, in terms of the level of maturity of both enterprises in general and manufacturing enterprises, showed groups of countries that should cooperate in digitalization. The exchange of good practices and joint application for European funds should become the standard of their activities in building the digital economy. It is also advisable for countries with a low level of digital economy to be supported by countries with a medium and high level of digital economy development. The solidarity of the European community requires broad cooperation, exchange of experience, technologies as well as technical, organizational and business solutions. Supporting less developed countries is a prerequisite for achieving very ambitious EU goals in both digitalization and ecology. In the case of the CEE countries, this requires broad assistance in building a knowledge-based economy, full utilization of economic potential, implementation of innovative solutions as well as research and development of new digital technologies.

The developed methodology, conducted research and obtained results significantly enrich the knowledge of the CEE countries, indicating their strengths and weaknesses. As mentioned earlier, at the current stage of the world’s economic development, the process of digitalization of enterprises is the basis for their development and determines their competitiveness. The consequence of delays in this regard will be a significant reduction in development prospects. Therefore, the development of these countries must be based on the digital economy.

In conclusion, the CEE countries belonging to the group of developing countries, through their presence in the EU have a great opportunity for dynamic economic and social growth. Their location and potential predispose them to such development, but the use of this opportunity largely depends on them. The rapid digitalization of the economy is just such an opportunity.
